# Supplementary material for: What can make things better for parents when babies need abdominal surgery in their first year of life? A qualitative interview study in the UK
Source: BMJ Open. 2018 Jun 30;8(6):e020921. doi: 10.1136/bmjopen-2017-020921 (PMC6042569; doi:10.1136/bmjopen-2017-020921)
Supplement: Author's manuscript [file bmjopen-2017-020921.draft_revisions.pdf]

BMJ Open

BMJ Open is committed to open peer review. As part of this commitment we make the peer review history of every article we publish publicly available.

When an article is published we post the peer reviewers' comments and the authors' responses online. We also post the versions of the paper that were used during peer review. These are the versions that the peer review comments apply to.

The versions of the paper that follow are the versions that were submitted during the peer review process. They are not the versions of record or the final published versions. They should not be cited or distributed as the published version of this manuscript.

BMJ Open is an open access journal and the full, final, typeset and author-corrected version of record of the manuscript is available on our site with no access controls, subscription charges or pay-per-view fees (http://bmjopen.bmj.com).

If you have any questions on BMJ Open's open peer review process please email info.bmjopen@bmj.com

BMJ Open

Making things better for parents when babies need surgery

Journal:	*BMJ Open*
Manuscript ID	bmjopen-2017-020921
Article Type:	Research
Date Submitted by the Author:	01-Dec-2017
Complete List of Authors:	Hinton, Lisa; University of Oxford, Health Experiences Research Group, Nuffield Department of Primary Care Health Sciencs<br>Locock, Louise; University of Aberdeen, Health Service Research<br>Long, Anna-May; University of Oxford, National Perinatal Epidemiology Unit<br>Knight, Marian; National Perinatal Epidemiology Unit
**Primary Subject Heading**:	Paediatrics
Secondary Subject Heading:	Surgery, Sociology, Qualitative research
Keywords:	Paediatric gastroenterology < GASTROENTEROLOGY, Paediatric intensive & critical care < INTENSIVE & CRITICAL CARE, QUALITATIVE RESEARCH, Pancreatic surgery < SURGERY

SCHOLARONE™
Manuscripts

Making things better for parents when babies need surgery

Hinton L, Locock L, Long A-M, Knight M.

Author information

Lisa Hinton, Nuffield Department of Primary Care Health Sciences, University of Oxford, Oxford. OX2 6GG, lisa.hinton@phc.ox.ac.uk, +44 (0)1865 289304

Louise Locock, Professor of Health Services Research, University of Aberdeen, Scotland AB25 2ZD

Marian Knight, NIHR Professor of Maternal and Child Population Health, National Perinatal Epidemiology Unit, University of Oxford OX3 7LF

Anna-May Long, Clinical Research Fellow in Paediatric Surgery

Word count 2814

For peer review only

ABSTRACT

(i) Objectives

To understand the experiences of parents of infants who required surgery early in life. To identify messages and training needs for the extended, clinical teams caring for these families – including paediatric surgeons, neonatologists, nurses, obstetricians, midwives and sonographers.

(ii) Setting

United Kingdom wide interview study, including England, Wales and Scotland.

(iii) Participants

In-depth narrative interviews were conducted with 44 parents who had a baby who underwent neonatal surgery. Conditions included those diagnosed antenatally (e.g. exomphalos, gastroschisis, congenital diaphragmatic hernia) or which developed postnatally (e.g. Hirschsprung's disease, necrotising enterocolitis). Interviews were video and audio recorded and analysed using a modified grounded-theory approach.

(iv) **Results**

While some parents reported experiencing excellent communication and felt they were listened to and involved by the care team, this was not always the case. Dealing with large, complex medical and surgical teams could result in conflicting messages, uncertainty and distress. Parents wanted information but also described being overwhelmed and wanting to distance themselves to maintain hope. Information and support from other parents in hospital and online groups was highly valued. Of particular concern was support for going home and caring for their baby after discharge; an open access policy for readmission offered a helpful safety net. The writings of Bourdieu could have resonance in interpreting

For peer review only

the experiences of parents as they enter the world of highly-technical neonatal medicine and surgery and the knowledge of the professionals who work in these environments.

(v) Conclusions

Listening to the experience of parents provides rich data to enhance clinical understandings on how to improve information and communication with parents, and ameliorate the deep and lasting distress and anxiety that some parents feel when their infants face early surgery.

Article Summary

Strengths and Limitations

- This is a UK based study that sought to explore the experiences of parents who have an infant requiring surgery in the first year of life. Interview studies in this field are rare and our study provides rich insights into the emotional and practical impacts of these experiences.
- While the interviews could not cover all possible conditions, they provide insights that may be generalisable across many different surgical procedures/medical conditions.
- A prenatal diagnosis of a congenital anomaly is associated with high levels of maternal psychological distress. Entering the complex environment of a neonatal intensive care unit is emotionally challenging for new parents
- Parents of children undergoing early surgery value being involved in care and decision-making; regular communication is an essential part of this.
- Clinicians should support and facilitate peer interactions both in person and through social media, as these are a vital source of support for many parents.

For peer review only

INTRODUCTION

The experiences of parents of infants who undergo surgery in the first year of life are rarely recorded. Even after discharge from hospital, these infants may require multiple outpatient visits to different specialist clinics and their ongoing care requirements can have significant effects on the quality of life of parents and existing children, as well as economic effects through lost work days.

Many of these infants will require surgery because of a gastrointestinal congenital anomaly, such as exomphalos, gastroschisis or Hirschsprung's disease. These conditions are under-researched[1], understanding of them is poor and interventions are rarely based on robust evidence. Routine sources of information are limited.[2] Outcomes for infants with rare anomalies are improving[3 4] and the need for rigorous research into surgical and clinical outcomes is paramount.[2] Alongside this, understanding the impact of these diagnoses and surgical treatments on parents and families is central to their long-term recovery.

There is little research on parent experiences of conditions that require early surgery. However, we can supplement this limited evidence base by drawing on research into parent experiences of congenital heart disease and surgery and more generally research about parents' experiences of neonatal intensive care units. The literature here points to the practical and psychological impact on families [5 6] and the importance of good communication between parents and staff.[7] Parents of children with congenital heart disease are at high risk for mental health morbidity, experiencing stress, depression, anxiety and traumatic stress responses [8-12] and the cost implications are profound.[13] Parents

For peer review only

experience difficulties with bonding and breastfeeding while their infants are in hospital [8 14 15 16] and their infants' complex needs after discharge.[17]

Mothers of infants in neonatal intensive care face emotional challenges as they seek to find a role for themselves in medically complex environments with perilously ill infants.[18] Wilkinson[19] and others have explored the contentious ethical issues facing those who care for critically ill infants and the extent to which parents should be involved in decision-making. Little is known about the relationship between quality of mother-child interaction and child development after major neonatal surgery or intensive care, but the parental mental health and familial environment are thought to be vital to the recovery and mental well-being of the infant.[20-22]

Theoretical framework

One approach to thinking about these experiences could be to draw on the writings of the French sociologist Pierre Bourdieu whose work focused on examining experiences of social class and the production of knowledge and power [23 24]. One of his key concepts was the notion of 'habitus', whereby individuals share their history and environment with others who have similar positions, producing a 'stylistic affinity' and where 'social identity is defined and asserted through difference'. Habitus is the way one unconsciously acts, interacts and behaves within the social world in a 'taken for granted' manner, according to socialised norms, traditions and unwritten rules of particular groups. Bourdieu also wrote about various forms of capital, qualities that establish social standing within a particular habitus or social setting. Capital is used to provide increased access to various resources within the field concerned (in this case neonatal care and surgery) and power is derived

For peer review only

from configurations of these types of capital[25]. Although his writings focused on class lifestyles and social identity, these ideas could have resonance in interpreting the experiences of parents as they enter the world of highly-technical neonatal medicine and surgery and the knowledge of the professionals who work in these environments[26].

The aim of this qualitative study was to: explore parents' experiences and perspectives of having a baby who needs surgery; identify the questions and problems that matter to parents during and after their pregnancy and infant's surgery; and identify the long-term impact on parents and families. In this article, we report on immediate actions that can be implemented to rapidly improve parent experience.

METHODS

We conducted interviews with 42 parents, all of whom had an infant who needed surgery in the first year of life. Parents were recruited from England, Scotland and Wales and included those for whom the experience was very recent through to parents whose son was 25 years old. Most parents' infants were still living, but we interviewed the parents of one boy who died aged seven months. Conditions included exomphalos, gastroschisis, congenital diaphragmatic hernia, Hirschsprung's disease, inguinal hernia, atresia (jejunal and oesophageal) and necrotising enterocolitis (see Table 1). Parents were interviewed either individually or in pairs. Recruitment was through support groups, paediatric surgeons, neonatal nurses, other specialists and word of mouth. We aimed for a maximum variation sample and continued interviews until thematic saturation was reached. All interviews were conducted by LH, in participants' own homes, or in a venue of their choice. Interviews were video or audio recorded and fully transcribed. We undertook analysis of the transcribed

For peer review only

interviews using an interpretive approach to thematic analysis.[27 28] Interview transcripts were coded by LH using a framework which was developed iteratively and reflected both anticipated and emergent themes. Coding reports were then analysed separately by LH and LL; any differences in interpretation were then discussed. MK and AML reviewed summarised findings and contributed a further clinical layer of analysis

Ethics Committee Approval: Ethics committee approval was given for this study by the Berkshire Ethics Committee, 09/H0505/66. All participants gave informed consent before taking part and have given written consent to their interview data being included in publications.

For peer review only

RESULTS

Some parents discovered there was a problem with their baby during antenatal scans, others not until after their baby had been born. Regardless of when the diagnosis came, parents of a baby who needs surgery faced many challenges over the ensuing months and years.

The care pathways for these infants were often long and complex. Setbacks were common, and at no point could clinical staff give parents any guarantees. The practical and emotional uncertainties were, therefore, huge. Living with uncertainty and worry was something parents had to get used to, at every stage.

"It was always a waiting game and tricky because you want to know the answer, 'When is my baby going to come home?'" (02, daughter with gastroschisis)

"We just don't know what road he is going to take." (20/21, son with Hirschsprung's disease)

Large and complex teams of staff across many different specialities were involved in looking after these infants over weeks, months and years. Our interviews highlighted some key steps that could improve the experience for parents. These do not so much focus on the surgery itself, but on the care and support parents are given before and after.

i) Communication

ii) Managing information and information overload

iii) Encouraging parents to seek out others (online, hospital groups)

iv) Help in finding a role

v) Preparing for going home

vi) Open access policy

Communication

Communication with the various health professionals looking after their baby was central to parents' experiences. These professionals spanned many disciplines – neonatologists, surgeons, but also paediatricians, intensive care nurses, specialist stoma nurses and other specialists further along, such as physiotherapists or nutritionists.

For peer review only - http://bmjopen.bmj.com/site/about/guidelines.xhtml

For peer review only

The babies were often very sick with a rare condition and receiving complex medical and surgical treatments. Parents often felt isolated and struggled to find information about their baby's condition. Communication about how their baby's progress and the treatment plan was therefore very important.

There were often extended periods of uncertainty as doctors were not able to answer parents' questions. While these periods were a challenge to parents, there were examples of good, clear communication which really helped at a stressful, frightening time.

i) Being kept up to date

Being kept up to date with their baby's care was important to parents, especially when plans changed. They valued the opportunity to ask questions repeatedly. ID25 said she was constantly asking questions but staff were very good at answering them all and reassuring her. (see Box 1)

ii) Communicating well

Although health professionals often had to deliver bad news, there were many examples of good practice in managing this well. These interactions were characterised by giving parents information in a clear, accessible manner, sometimes supported by drawing diagrams, but without patronising/'dumbing down'. Parents appreciated doctors and nurses taking time to answer their questions, not making them feel that any question was a silly one.

For peer review only

iii) Conveying expertise

When infants were in hospital for long periods, relationships of mutual respect between parents and healthcare professionals were hugely valued. As one mother explained, “you are handing over the most precious thing to this person who is going to put them under anaesthetic and disappear into an operating theatre with them and you hope their gonna come out the other side. So you have to have that level of assurance.” She and her husband were reassured by their surgeon’s “confidence not arrogance”. Others talked about the importance of trust and feeling as though their baby was in expert hands, cared for by clinicians who were knowledgeable about their condition.

iv) Being listened to and feeling part of the team

While parents recognised that they relied on doctors’ expertise and judgement in planning their baby’s care, they really valued feeling involved in their baby’s care – “part of it, part of that team”.

Managing information and information overload

Understanding the diagnosis and possible journey ahead can be overwhelming for parents. Parents often felt powerless, both during pregnancy and once their baby had been born. Information – about the condition and procedures but also other parent experiences – was crucial in helping parents come to terms with the diagnosis. Seeking out information played a central role, helping parents feel they were actively doing something for their baby and

For peer review only

family. ID01's son had exomphalos. Seeking information felt like the only thing she and her husband could do. *"We were powerless to change anything other than lots of information".*

However, there were also dangers of information overload. Parents could find it hard to take on board a great deal of complex information at once, and needed time to understand and process what was happening to their baby. On the whole, parents said the information given by hospitals was not sufficient and they supplemented it with their own background reading and research. Pointing parents in the direction of trusted sources of information would have been a key easy action to help them.

Encouraging parents to seek out others

Finding support from other parents who had been through similar experiences (either online or face to face) was described as a crucial factor in helping parents cope and understand more about their baby's diagnosis. Staff can encourage and facilitate this through introducing parents to each other, or suggesting online support groups. One unit had established a popular and thriving Facebook support group for parents of babies with Hirschsprung's disease.

Helping parents find a role

Parents often described how "useless" or "helpless" they felt not being able to do anything for their baby while in hospital. Yet when they went home they were going to become experts in their baby's care. Helping them bridge this gap was an area where staff could make a real impact.

For peer review only

In the early days, spending time at their baby's side was all many parents felt they could do. They looked for small ways they could regain a sense of control and get involved in their baby's care. While it was not always practical in critical care environments, parents appreciated helping with feeding, bathing, singing and talking to their baby where possible. After three weeks, ID02 was able to hold her daughter for the first time, "*it was like Christmas*". Supporting mothers to either breastfeed or, more often, express milk for their baby, was another way of parents bonding and being involved in their baby's care. Some parents described how they were supported and encouraged to become "experts" in aspects of their baby's daily care – changing dressings or stoma bags. Activities like these were key to bridging the gap between hospital and home.

Preparing to go home

After weeks or months in hospital, being able to bring their baby home was a huge step for many parents. While a positive sign that their baby was on the mend, it could also be daunting to leave the "safety net" of hospital and clinical expertise. Some parents described handover or normalisation programmes as very helpful in preparing them technically and emotionally for caring for their infant at home. Parents were trained in some of the skills they would need (wound management, stoma care, first aid, resuscitation) and given the opportunity to 'room in' with their baby for a few nights before going home.

Open access policy

Accessing hospitals and specialists after their baby had been sent home could be challenging for parents, especially if home was a distance from the specialist surgical centre. When problems came up, as they often did, it was hard to know where to take their baby. So being

For peer review only - http://bmjopen.bmj.com/site/about/guidelines.xhtml

given open or emergency access back to the team who had looked after their baby, without having to go through A&E first, was really valued by parents.

DISCUSSION

This study adds valuable insights into the support and information needs of parents when their baby requires neonatal surgery. These surgical journeys are often a long haul for baby and parents. Our study highlights the importance of clear and ongoing communication between parents and the often large, multi-disciplinary teams caring for their infant and points to immediate actions that can improve parent experiences and outcomes. Parents value being listened to, encouraged to be a part of the care team and supported in finding a role for themselves. They need information from the professionals caring for their infant but also recommendations of good sources of information to supplement their knowledge, while being mindful of information overload. For some, encouraging them to seek support from other parents is of great value.

Previous work exploring parents' experiences of having an infant in NICU or surgery has highlighted the importance of good communication and emotional support, giving parents support at handover for surgery and pointed to the impact of scanty and infrequent information giving.[7 29] Parents experiences are not only affected by their baby's diagnosis and care, but also the ways in which services are organised to support families to navigate the systems and maintain a new 'second campus' as they look after their baby and continue their lives outside the hospital.[30]

For peer review only

Involving and supporting parents is increasingly recognised as an important component of providing holistic paediatric care and can help with the uncertainty of parenting an infant during critical illness. Parent experiences of NICU point to the vital role that neonatal nurses have in engaging with mothers and the importance of that relationship.[30] Aagaard[5] discussed the importance of trying to strengthen maternal competence, and suggested parent-nurse chat as a communication strategy. Our findings provide examples of the ways in which encouraging parents to be involved with infant can be achieved.

Others have described the role of the expert parent [30] and home monitoring programmes after cardiac surgery.[17 31] Studies have indicated that parents are not adequately prepared for discharge and are not well equipped to recognise deterioration in their child. The positive examples of being prepared for discharge and supported in becoming the primary carer offer insights into how parents might be supported to manage this transition.

Bourdieu's theoretical concepts of habitus and capital are helpful in exploring and unpacking the experiences of parents in these highly technical environments and the value they place on communication, finding their own role and seeking out other parents. They travel a journey from being newcomers in an environment (neonatal/surgical unit) where they are unfamiliar with the socialised norms. They have little capital and no idea how to 'play the game' (what Bourdieu termed the logic of practice) and negotiate the field. There is initially an unequal balance of power. But over time, assisted by good communication from the health professionals and the networks of support and information provided by other parents, parents develop confidence and competency which Bourdieu might have

For peer review only

interpreted as social capital. By the time they take their infants home these parents are well on the way to becoming technical experts in their baby's illness and care.

STRENGTHS AND LIMITATIONS

This is a UK based study that sought to explore the experiences of parents who have an infant requiring surgery in the first year of life. Interview studies in this field are rare and our study provides rich insights into the emotional and practical impacts of these experiences. While the interviews could not cover all possible conditions, they provide insights that may be generalisable across many different surgical procedures/medical conditions.

CONCLUSION

As babies recover, the potential for parental expertise grows. Our study highlights the vital role of information, both about the condition and about the infant's progress. Health care staff could discuss or direct parents to information sources, seek to empower parents through involving them with their infant's care where possible, and link them with other parents.

A Contributorship statement The study was conceived by MK. All interviews were collected by LH. Analysis was undertaken by LH and LL, with input from MK and AM. LH wrote the article, with input from the other authors.

B Competing Interests Authors declare no competing interests

C Funding: This study was funded through a National Institute for Health Research (NIHR) Professorship award to Marian Knight (NIHR-RP-011-032). The views expressed are those of the author(s) and not necessarily those of the NHS, the NIHR or the Department of Health and by the National Institute for Health Research Biomedical Research Centre, Oxford, grant BRC-1215-20008 to the Oxford University Hospitals NHS Foundation Trust and the University of Oxford.

For peer review only

D Data Sharing statement Participants were invited to review their transcript and mark any sections that they did not want used before transferring copyright to the University of Oxford for use in research, teaching, publications and broadcasting. These carefully anonymised transcripts form part of a University of Oxford archive which is available to other bona fide research teams for secondary analysis. All authors had access to the all of the data in the study and take responsibility for the integrity of the data and the accuracy of the data analysis.

For peer review only

REFERENCES

1. Allin BSR, Tse WHW, Marven S, et al. Challenges of Improving the Evidence Base in Smaller Surgical Specialties, as Highlighted by a Systematic Review of Gastroschisis Management. *Plos One* 2015;10(1) doi: ARTN e0116908
10.1371/journal.pone.0116908
2. Allin B, Ross A, Marven S, et al. Development of a core outcome set for use in determining the overall success of gastroschisis treatment. *Trials* 2016;17 doi: ARTN 360
10.1186/s13063-016-1453-7
3. Lam HS, Sit T, Chau CL, et al. Attitudes of Parents and Health Care Workers to Major Surgery for High-Risk Preterm Infants. *The Journal of Pediatrics* 2016;177(Supplement C):78-83.e3. doi: https://doi.org/10.1016/j.jpeds.2016.06.071
4. Tam PKH, Chung PHY, St Peter SD, et al. Advances in paediatric gastroenterology. *Lancet* 2017;390(10099):1072-82.
5. Aagaard H, Hall EOC. Mothers' Experiences of Having a Preterm Infant in the Neonatal Care Unit: A Meta-Synthesis. *Journal of Pediatric Nursing* 2008;23(3):e26-e36. doi: https://doi.org/10.1016/j.pedn.2007.02.003
6. Wei H, Roscigno CI, Hanson CC, et al. Families of children with congenital heart disease: A literature review. *Heart Lung* 2015;44(6):494-511. doi: 10.1016/j.hrtlng.2015.08.005
7. Wigert H, Dellenmark MB, Bry K. Strengths and weaknesses of parent-staff communication in the NICU: a survey assessment. *Bmc Pediatr* 2013;13 doi: Artn 71
10.1186/1471-2431-13-71
8. Jordan B, Franich-Ray C, Albert N, et al. Early mother-infant relationships after cardiac surgery in infancy. *Arch Dis Child* 2014;99(7):641-5. doi: 10.1136/archdischild-2012-303488
9. Woolf-King SE, Anger A, Arnold EA, et al. Mental Health Among Parents of Children With Critical Congenital Heart Defects: A Systematic Review. *J Am Heart Assoc* 2017;6(2) doi: ARTN e004862
10.1161/JAHA.116.004862
10. Aite L, Bevilacqua F, Zaccara A, et al. Seeing Their Children in Pain: Symptoms of Posttraumatic Stress Disorder in Mothers of Children with an Anomaly Requiring Surgery at Birth. *Am J Perinat* 2016;33(8):770-75. doi: 10.1055/s-0036-1572543
11. Le Gouez M, Alvarez L, Rousseau V, et al. Posttraumatic Stress Reactions in Parents of Children Esophageal Atresia. *Plos One* 2016;11(3) doi: ARTN e0150760
10.1371/journal.pone.0150760
12. Kaasen A, Helbig A, Malt UF, et al. Acute maternal social dysfunction, health perception and psychological distress after ultrasonographic detection of a fetal structural anomaly. *BJOG* 2010;117(9):1127-38. doi: 10.1111/j.1471-0528.2010.02622.x
13. Connor JA, Kline NE, Mott S, et al. The Meaning of Cost for Families of Children With Congenital Heart Disease. *J Pediatr Health Car* 2010;24(5):318-25. doi: 10.1016/j.pedhc.2009.09.002
14. Froh EB, Deatrick JA, Curley MAQ, et al. Making Meaning of Pumping for Mothers of Infants With Congenital Diaphragmatic Hernia. *Jognn-J Obst Gyn Neo* 2015;44(3):439-49. doi: 10.1111/1552-6909.12564
15. Ainsworth MDB, M; Waters, E. . Patterns of attachment: a psychological study of the strange situation. New Jersey: Erlbaum 1978.

For peer review only

16. Bowlby J. The nature of the child's tie to the mother. *International Journal of Psychoanalysis* 1958;38(9):350-72.
17. Tregay J, Brown KL, Crowe S, et al. Signs of deterioration in infants discharged home following congenital heart surgery in the first year of life: a qualitative study. *Arch Dis Child* 2016;101(10):902-8. doi: 10.1136/archdischild-2014-308092
18. Lupton D, Fenwick J. 'They've forgotten that I'm the mum': constructing and practising motherhood in special care nurseries. *Soc Sci Med* 2001;53(8):1011-21. doi: Doi 10.1016/S0277-9536(00)00396-8
19. Wilkinson D. Death or Disability? The 'Carmentis Machine' and decision-making for critically ill children. Oxford: Oxford University Press 2013.
20. Kubota A, Yamakawa S, Yamamoto E, et al. Major neonatal surgery: psychosocial consequence of the patient and mothers. *J Pediatr Surg* 2016;51(3):364-67. doi: 10.1016/j.jpedsurg.2015.09.017
21. Laing S, McMahon C, Ungerer J, et al. Mother–child interaction and child developmental capacities in toddlers with major birth defects requiring newborn surgery. *Early Human Development* 2010;86(12):793-800. doi: https://doi.org/10.1016/j.earlhumdev.2010.08.025
22. Kratochvil MS, Robertson CMT, Kyle JM. Parents' View of Parent-Child Relationship Eight Years After Neonatal Intensive Care. *Social Work in Health Care* 1992;16(1):95-118. doi: 10.1300/J010v16n01_06
23. Huppatz K. Pierre Bourdieu: Health Lifestyles, the Family and Social Class. In: Collyer F, ed. Palgrave Handbook of Social Theory in Health, Illness and Medicine. Basingstoke: Palgrave Macmillan 2015:370-88.
24. Webb J, Schirato, T, Danaher, G, . Understanding Bourdieu. London: Sage 2002.
25. Bourdieu P. What makes a social class? On the theoretical and practical existence of groups. *Berkeley J Sociol* 1987;32:1-17.
26. Bourdieu P. Distinction: A Critique of the Judgement of Taste. London: Routledge 1984.
27. Glaser BS, Strauss A. The Discovery of Grounded Theory. New York: Adine Publishing 1967.
28. Green JT, Thorogood N. Qualitative Methods for Health Research. London: Sage 2004.
29. Wei H, Roscigno CI, Swanson KM, et al. Parents' experiences of having a child undergoing congenital heart surgery: An emotional rollercoaster from shocking to blessing. *Heart & Lung: The Journal of Acute and Critical Care* 2016;45(2):154-60. doi: https://doi.org/10.1016/j.hrtlng.2015.12.007
30. Kosta L, Harms L, Franich-Ray C, et al. Parental experiences of their infant's hospitalization for cardiac surgery. *Child Care Hlth Dev* 2015;41(6):1057-65. doi: 10.1111/cch.12230
31. Gaskin KL, Barron DJ, Daniels A. Parents' preparedness for their infants' discharge following first-stage cardiac surgery: development of a parental early warning tool. *Cardiol Young* 2016;26(7):1414-24. doi: 10.1017/S1047951116001062

For peer review only

For peer review only

Table of Participants

Table 1 Participants

Condition	Identifier	Sex of child	Child's age at interview
Exomphalos	01	m	18 months
	02	f	6 years
	03	f	8 years
	04/05	f	8 years
	12/13	m	25 years
	16/17	m	13 years
	19	f	16 months
	41/42	m	Died age 7 months
Necrotising Enterocolitis	08	m	9 months
	09	m	11 months
	23	m	6 months
Hernia	26/27	f	9 months
Congenital Diaphragmatic Hernia	10/11	m	9 months
	15	m	5 years
	24	m	9 years
Hirschprung's Disease	18	f	1 year
	20/21	m	4 months
	22	m	16 months
	28/29	m	19 months
	39/40	m	5 years old
	43/44	m	5 years old
Gastroschisis	30	m	7 ½ months
	31	m	4 years
	32	f	3 years
	33/34	m	3 months
	36	f	19 months
	37	f	6 years
Short Bowel	35	f	7 years
Jejunal atresia	25	f	4 months
Oesophageal Atresia with trachea-oesophageal fistula	38	m	19 months
Undiagnosed	14	m	6 months

BOX 1

Communication

"If you're in the dark that's when people would worry [….] as long as you, you know, either good or bad, what was going on and a lot of the things you ask them they don't know the answer because it's a time will tell kind of answer, but so long as someone tells you the time will tell sort of answer you've got an answer." (ID25, daughter with jejunal atresia)

Being kept up to date

"[I] probably annoyed the hell out of them, but wanted to know, OK, when he does this what is his next step, what has progressed to, how long will he be doing that and how long will it take and things." (ID15, son with CDH)

Communicating well

"I quite like things in a lot of detail so that when doctors are talking to you sometimes they can do stupid talk because they don't know how much you know medically, but I like to hear all of it and I don't want broken down terms, I want medical terms because then I can go and look up what the medical terms are, see what it's all about." (ID25, daughter with jejunal atresia)

Conveying expertise

"They've got this under control, they really know what they are doing." (ID26, daughter with ovarian hernia)

"I will trust you to the ends of the world" (ID38, describing the neonatal nurse who looked after her son with Oesophageal Atresia with trachea-oesophageal fistula)

Being listened to and feeling part of the team

"They've been very understanding and patient and I think recognising as well our role as parents and, that we, you know, have opinions and experiences, and not devaluing what we've learnt from other places as well." (ID01, son with exomphalos)

"I would say 99.9% of the time we've been treated really well, like human beings, not just numbers, and that has made an enormous difference to the experience as a family." (ID03, daughter with exomphalos)

Encouraging parents to seek out others

"It just makes it a bit real, you know, that you aren't the only one". (ID22, son with Hirschsprung's disease)

"*Find parents, my absolute number one thing."* (ID01, son with exomphalos)

For peer review only

"That was my number one support during that time with all these other mums who'd gone through it." (ID02, daughter with gastroschisis).

Helping parents find a role

"I think you're so wrapped up in the medical needs of your baby and hoping that they're going to be alive at the end of it that I just…hadn't anticipated that the little things would be the things that matter, it would be the 'mummy' things that had been so easy to do with my other children, they would be the things that would matter that I missed out on." (ID19, daughter with exomphalos).

Preparing to go home

"You're totally encouraged to do as much care for your baby as you can. It's your baby, they're there to support you and to medically [um] jump in if, if need be, but don't expect that they're there to feed and care for your baby because that's your job." (ID34, son with gastroschisis)

For peer review only

For peer review only - http://bmjopen.bmj.com/site/about/guidelines.xhtml

BOX 1

Communication

"If you're in the dark that's when people would worry [....] as long as you, you know, either good or bad, what was going on and a lot of the things you ask them they don't know the answer because it's a time will tell kind of answer, but so long as someone tells you the time will tell sort of answer you've got an answer." (ID25, daughter with jejunal atresia)

Being kept up to date

"[I] probably annoyed the hell out of them, but wanted to know, OK, when he does this what is his next step, what has progressed to, how long will he be doing that and how long will it take and things." (ID15, son with CDH)

Communicating well

"I quite like things in a lot of detail so that when doctors are talking to you sometimes they can do stupid talk because they don't know how much you know medically, but I like to hear all of it and I don't want broken down terms, I want medical terms because then I can go and look up what the medical terms are, see what it's all about." (ID25, daughter with jejunal atresia)

Conveying expertise

"They've got this under control, they really know what they are doing." (ID26, daughter with ovarian hernia)

"I will trust you to the ends of the world" (ID38, describing the neonatal nurse who looked after her son with Oesophageal Atresia with trachea-oesophageal fistula)

Being listened to and feeling part of the team

"They've been very understanding and patient and I think recognising as well our role as parents and, that we, you know, have opinions and experiences, and not devaluing what we've learnt from other places as well." (ID01, son with exomphalos)

"I would say 99.9% of the time we've been treated really well, like human beings, not just numbers, and that has made an enormous difference to the experience as a family." (ID03, daughter with exomphalos)

Encouraging parents to seek out others

"It just makes it a bit real, you know, that you aren't the only one". (ID22, son with Hirschsprung's disease)

"*Find parents, my absolute number one thing."* (ID01, son with exomphalos)

For peer review only

"That was my number one support during that time with all these other mums who'd gone through it." (ID02, daughter with gastroschisis).

Helping parents find a role

"I think you're so wrapped up in the medical needs of your baby and hoping that they're going to be alive at the end of it that I just…hadn't anticipated that the little things would be the things that matter, it would be the 'mummy' things that had been so easy to do with my other children, they would be the things that would matter that I missed out on." (ID19, daughter with exomphalos).

Preparing to go home

"You're totally encouraged to do as much care for your baby as you can. It's your baby, they're there to support you and to medically [um] jump in if, if need be, but don't expect that they're there to feed and care for your baby because that's your job." (ID34, son with gastroschisis)

For peer review only

Table of Participants

Table 1 Participants

Condition	Identifier	Sex of child	Child's age at interview
Exomphalos	01	m	18 months
	02	f	6 years
	03	f	8 years
	04/05	f	8 years
	12/13	m	25 years
	16/17	m	13 years
	19	f	16 months
	41/42	m	Died age 7 months
Necrotising Enterocolitis	08	m	9 months
	09	m	11 months
	23	m	6 months
Hernia	26/27	f	9 months
Congenital Diaphragmatic Hernia	10/11	m	9 months
	15	m	5 years
	24	m	9 years
Hirschprung's Disease	18	f	1 year
	20/21	m	4 months
	22	m	16 months
	28/29	m	19 months
	39/40	m	5 years old
	43/44	m	5 years old
Gastroschisis	30	m	7 ½ months
	31	m	4 years
	32	f	3 years
	33/34	m	3 months
	36	f	19 months
	37	f	6 years
Short Bowel	35	f	7 years
Jejunal atresia	25	f	4 months
Oesophageal Atresia with trachea-oesophageal fistula	38	m	19 months
Undiagnosed	14	m	6 months

For peer review only

BMJ Open

What can make things better for parents when babies need abdominal surgery in their first year of life? A qualitative interview study in the United Kingdom

Journal:	*BMJ Open*
Manuscript ID	bmjopen-2017-020921.R1
Article Type:	Research
Date Submitted by the Author:	06-Feb-2018
Complete List of Authors:	Hinton, Lisa; University of Oxford, Health Experiences Research Group, Nuffield Department of Primary Care Health Sciencs<br>Locock, Louise; University of Aberdeen, Health Service Research<br>Long, Anna-May; University of Oxford, National Perinatal Epidemiology Unit<br>Knight, Marian; National Perinatal Epidemiology Unit
**Primary Subject Heading**:	Paediatrics
Secondary Subject Heading:	Surgery, Sociology, Qualitative research
Keywords:	Paediatric gastroenterology < GASTROENTEROLOGY, Paediatric intensive & critical care < INTENSIVE & CRITICAL CARE, QUALITATIVE RESEARCH, Pancreatic surgery < SURGERY

SCHOLARONE™
Manuscripts

What can make things better for parents when babies need abdominal surgery in their first year of life? A qualitative interview study in the United Kingdom

Hinton L, Locock L, Long A-M, Knight M.

Author information

Lisa Hinton, DPhil, Nuffield Department of Primary Care Health Sciences, University of Oxford, Oxford. OX2 6GG, lisa.hinton@phc.ox.ac.uk, +44 (0)1865 289304

Louise Locock, Professor of Health Services Research, University of Aberdeen, Scotland AB25 2ZD

Marian Knight, NIHR Professor of Maternal and Child Population Health, National Perinatal Epidemiology Unit, University of Oxford OX3 7LF

Anna-May Long, Clinical Research Fellow in Paediatric Surgery

Word count [4536]

For peer review only

For peer review only

For peer review only - http://bmjopen.bmj.com/site/about/guidelines.xhtml

ABSTRACT

(i) Objectives

To understand the experiences of parents of infants who required surgery early in life. To identify messages and training needs for the extended clinical teams caring for these families – including paediatric surgeons, neonatologists, nurses, obstetricians, midwives and sonographers.

(ii) Setting

United Kingdom wide interview study, including England, Wales and Scotland.

(iii) Participants

In-depth semi-structured narrative interviews were conducted with 44 parents who had a baby who underwent early surgery. Conditions included those diagnosed antenatally (e.g. exomphalos, gastroschisis, congenital diaphragmatic hernia) or which were detected postnatally (e.g. Hirschsprung's disease, necrotising enterocolitis). Interviews were video and audio recorded and analysed using a modified grounded-theory approach.

(iv) **Results**

While some parents reported experiencing excellent communication and felt they were listened to and involved by the care team, this was not always the case. Dealing with large, complex medical and surgical teams could result in conflicting messages, uncertainty and distress. Parents wanted information but also described being overwhelmed and wanting to distance themselves to maintain hope. Information and support from other parents in hospital and online groups was highly valued. Of particular concern was support when going home and caring for their baby after discharge; an open access policy for readmission offered a helpful safety net.

(v) Conclusions

For peer review only

Listening to the experience of parents provides rich data to enhance clinical understandings on how to improve information and communication with parents, and ameliorate the deep and lasting distress and anxiety that some parents feel when their infants face early surgery. We suggest that the writings of Bourdieu could have resonance in interpreting the experiences of parents as they enter the world of highly-technical neonatal medicine and surgery and the knowledge of the professionals who work in these environments.

Article Summary

Strengths and Limitations

- This is a UK based study that sought to explore the experiences of parents who have an infant requiring surgery in the first year of life.
- Interview studies in this field are rare and our study provides rich insights into the emotional and practical impacts of these experiences.
- While the interviews could not cover all possible conditions, they provide insights that may be generalisable across many different surgical procedures/medical conditions.

INTRODUCTION

The experiences of parents of infants who undergo surgery in the first year of life are rarely recorded. Even after discharge from hospital, these infants may require multiple outpatient visits to different specialist clinics and their ongoing care requirements can have significant effects on the quality of life of parents and existing children, as well as economic effects through lost work days.

For peer review only

Many of these infants will require surgery because of a gastrointestinal congenital anomaly, such as exomphalos, gastroschisis or Hirschsprung's disease. These conditions are under-researched[1], understanding of them is poor and interventions are rarely based on robust evidence. Routine sources of information are limited.[2] Outcomes for infants with rare anomalies are improving[3 4] and the need for rigorous research into surgical and clinical outcomes is paramount.[2] Alongside this, understanding the impact of these diagnoses and surgical treatments on parents and families is central to their long-term recovery.

This study was conducted as an adjunct to the British Association of Paediatric Surgeons Congenital Anomaly Surveillance System (BAPS-CASS) and was focused on parents' experiences of abdominal surgery. There is little research on parent experiences of conditions that require early abdominal surgery. However, we can supplement this limited evidence base by drawing on research into parent experiences of congenital heart disease and surgery and more generally research about parents' experiences of neonatal intensive care units. The literature here points to the practical and psychological impact on families [5 6] and the importance of good communication between parents and staff.[7] Parents of children with congenital heart disease are at high risk for mental health morbidity, experiencing stress, depression, anxiety and traumatic stress responses [8-12] and the cost implications are profound.[13] Parents experience difficulties with bonding and breastfeeding while their infants are in hospital [8 14 15 16]and their infants' complex needs after discharge.[17]

Mothers of infants in neonatal intensive care face emotional challenges as they seek to find a role for themselves in medically complex environments with perilously ill infants.[18] Wilkinson[19] and others have explored the contentious ethical issues facing those who care

For peer review only

for critically ill infants and the extent to which parents should be involved in decision-making. Little is known about the relationship between quality of mother-child interaction and child development after major neonatal surgery or intensive care, but the parental mental health and familial environment are thought to be vital to the recovery and mental well-being of the infant.[20-22]

Theoretical framework

One approach to thinking about these experiences could be to draw on the writings of the French sociologist Pierre Bourdieu whose work focused on examining experiences of social class and the production of knowledge and power [23 24]. One of his key concepts was the notion of 'habitus', whereby individuals share their history and environment with others who have similar positions, producing a 'stylistic affinity' and where 'social identity is defined and asserted through difference'. Habitus is the way one unconsciously acts, interacts and behaves within the social world in a 'taken for granted' manner, according to socialised norms, traditions and unwritten rules of particular groups[25]. Bourdieu identifies various forms of 'capital', economic, social and cultural resources that establish social standing within a particular habitus or social setting. Power is derived from configurations of these types of capital[26] within a given field (in this case neonatal care and surgery) and. Although his writings focused on class lifestyles and social identity, these ideas could have resonance in interpreting the experiences of parents as they enter the world of highly-technical neonatal medicine and surgery and the knowledge of the professionals who work in these environments[27].

For peer review only - http://bmjopen.bmj.com/site/about/guidelines.xhtml

The aim of this qualitative study was to: explore parents' experiences and perspectives of having a baby who needs early surgery; identify the questions and problems that matter to parents during and after their pregnancy and infant's surgery; and identify the long-term impact on parents and families. In this article, we report on the period immediately before and after surgery, and suggest actions that can be implemented to rapidly improve parent experience. Findings on the longer-term impacts of these experiences will be reported elsewhere.

METHODS

We conducted interviews with 44 parents, all of whom had an infant who needed surgery in the first year of life. Two parents did not return their copyright form so their data is not included in our analysis.

Participants

Parents were recruited from England, Scotland and Wales. We interviewed parents whose infant had required surgery in their first year of life. But we sought parents whose infant or child was now a wide variety of differing ages, as we were keen to capture a long term, not only a recent, perspective on the experiences. Therefore participants included those for whom the experience was very recent through to parents whose son was 25 years old. Most parents' infants were still living, but we interviewed the parents of one boy who died aged seven months. We interviewed some mothers individually and eleven couples (mother and father). Conditions that their infants had been diagnosed with included exomphalos, gastroschisis, congenital diaphragmatic hernia, Hirschsprung's disease, inguinal hernia, atresia (jejunal and oesophageal) and necrotising enterocolitis (see Table 1). Some

For peer review only - http://bmjopen.bmj.com/site/about/guidelines.xhtml

conditions were diagnosed antenatally, others developed after their baby was born. Some conditions were congenital, others developed as a result of premature birth or spontaneously (see Table 1). All infants had their surgery in the first year of life, although some went on to have multiple operations. Several spent extended periods of time in neonatal intensive care units (see Table 1).

Interviews

Parents were interviewed either individually or in pairs, depending on preference. We interviewed eleven fathers, but none chose to be interviewed on their own. Recruitment was through support groups, paediatric surgeons, neonatal nurses, other specialists and word of mouth. We aimed for a maximum variation sample and continued interviews until thematic saturation was reached. All interviews were conducted by LH, a social scientist, in participants' own homes, or in a venue of their choice using a semi-structured narrative approach[28]. Interviewees were asked an open-ended question at the start of the interview, followed up by a semi-structured section to capture themes of particular interest. The interview schedule was developed by co-authors in consultation with the study advisory panel and a group of parent advisors. All interviews were audio recorded, and where consent was given video recorded to facilitate dissemination on the patient experience website, healthtalk.org. Parents were offered a two-stage consent and copyright approval process. They signed a consent form before the interview started and were subsequently sent a copy of their transcript to approve before signing a copyright form agreeing to their data being included in the analysis and excerpts from their interviews included in dissemination, including peer review articles, education materials and on the healthtalk.org website. Those who wanted to remain anonymous could choose to have only audio or

For peer review only

written-only excerpts of their interviews included, and a pseudonym. Interviews lasted from between 75 minutes and 3 and a half hours. All interviews were fully transcribed. We undertook analysis of the transcribed interviews using an interpretive approach to thematic analysis.[29 30] Interview transcripts, not videos, were coded by LH with support from NVIVO analysis software, using a framework which was developed iteratively and reflected both anticipated and emergent themes. Coding reports were then analysed separately by LH and LL using a modified grounded theory approach, incorporating constant comparison and exploration of deviant cases, allowing the data to be grouped into themes and all cases to be examined to ensure all the manifestations of each theme were accounted for and compared[31] and we were satisfied we had reached data saturation with no new constructs emerging. [30]Any differences in interpretation were then discussed. MK and AML reviewed summarised findings and contributed a further clinical layer of analysis

Ethics Committee Approval: Ethics committee approval was given for this study by the Berkshire Ethics Committee, 09/H0505/66. All participants gave informed consent before taking part and have given written consent to their interview data being included in publications.

RESULTS

Some parents discovered there was a problem with their baby during antenatal scans, others not until after their baby had been born. Regardless of when the diagnosis came, parents of a baby who needs surgery faced many challenges over the ensuing months and years.

For peer review only - http://bmjopen.bmj.com/site/about/guidelines.xhtml

The care pathways for these infants were often long and complex. Setbacks were common, and at no point could clinical staff give parents any guarantees. The practical and emotional uncertainties were, therefore, huge. Living with uncertainty and worry was something parents had to get used to, at every stage. Many parents described a lasting distress and anxiety.

"It was always a waiting game and tricky because you want to know the answer, 'When is my baby going to come home?'" (ID02, daughter with gastroschisis)

"*We just don't know what road he is going to take."* (ID20, mother, son with Hirschsprung's disease)

"My anxiety levels have been awful really it's been hard for everybody because I've been up and down emotionally". (ID23, son with NEC)

Large and complex teams of staff across many different specialities were involved in looking after these infants over weeks, months and years. Our interviews highlighted some key steps that could improve the experience for parents. These do not so much focus on the surgery itself, but on the care and support parents are given before and after.

i) Communication
ii) Managing information and information overload
iii) Encouraging parents to seek out others (online, hospital groups)
iv) Help in finding a role
v) Preparing for going home
vi) Open access policy

Communication

Communication with the various health professionals looking after their baby was central to parents' experiences. These professionals spanned many disciplines – neonatologists, surgeons, but also paediatricians, intensive care nurses, specialist stoma nurses and other allied health professionals further along, such as physiotherapists or nutritionists.

The babies were often very sick with a rare condition and receiving complex medical and surgical treatments. Parents often felt isolated and struggled to find information about their baby's condition. Communication about how their baby's progress and the treatment plan was therefore very important.

"If you're in the dark that's when people would worry [….] as long as you, you know, either good or bad, what was going on and a lot of the things you ask them they don't know the answer because it's a time will tell kind of answer, but so long as someone tells you the time will tell sort of answer you've got an answer." (ID25, daughter with jejunal atresia)

There were often extended periods of uncertainty as doctors were not able to answer parents' questions. While these periods were a challenge to parents, there were examples of good, clear communication which really helped at a stressful, frightening time.

i) Being kept up to date

Being kept up to date with their baby's care was important to parents, especially when plans changed. They valued the opportunity to ask questions repeatedly. ID25 said she was

For peer review only - http://bmjopen.bmj.com/site/about/guidelines.xhtml

constantly asking questions but staff were very good at answering them all and reassuring her.

"[I] probably annoyed the hell out of them, but wanted to know, OK, when he does this what is his next step, what has progressed to, how long will he be doing that and how long will it take and things." (ID15, son with CDH)

The nurses were good as well at feeding back what the doctors had said and also if you had any questions I felt comfortable saying look can you ask about this and let me know what they say, so. (ID30, son with gastroschisis)

ii) Communicating well

Although health professionals often had to deliver bad news, there were many examples of good practice in managing this well. These interactions were characterised by giving parents information in a clear, accessible manner, sometimes supported by drawing diagrams, but without patronising/'dumbing down'. Parents appreciated doctors and nurses taking time to answer their questions, not making them feel that any question was a silly one.

M:And went through them with the consultant and actually again that was another thing that he very patiently sat and went through every single one of those questions, however stupid they were, you know.

***F:** How many times he'd heard them, no doubt.*

***M:** Yeah and it didn't.*

***F:** He wasn't fazed, he wasn't awkward he was just 'Okay, let's do it'.*
(ID 43/44, mother and father, son with Hirschsprung's disease)

"I quite like things in a lot of detail so that when doctors are talking to you sometimes they can do stupid talk because they don't know how much you know medically, but I like to hear all of it and I don't want broken down terms, I want medical terms because then I can go and look up what the medical terms are, see what it's all about." (ID25, daughter with jejunal atresia)

For peer review only

iii) Conveying expertise

When infants were in hospital for long periods, relationships of mutual respect between parents and healthcare professionals were hugely valued. As one mother explained, “you are handing over the most precious thing to this person who is going to put them under anaesthetic and disappear into an operating theatre with them and you hope their gonna come out the other side. So you have to have that level of assurance.” She and her husband were reassured by their surgeon’s “confidence not arrogance”. Others talked about the importance of trust and feeling as though their baby was in expert hands, cared for by clinicians who were knowledgeable about their condition.

“They’ve got this under control, they really know what they are doing…… In a way it’s silly thinking that now because of course they know what they’re doing these are highly trained professionals but in that moment that’s what you want to hear cos you just want to, you’re handing over your child so you want someone to be right on it don’t you and he was.” (ID26, father, daughter with iguinal hernia)

iv) Being listened to and feeling part of the team

While parents recognised that they relied on doctors’ expertise and judgement in planning their baby’s care, they really valued feeling involved in their baby’s care – “part of it, part of that team”.

“They’ve been very understanding and patient and I think recognising as well our role as parents and, that we, you know, have opinions and experiences, and not devaluing what we’ve learnt from other places as well.” (ID01, son with exomphalos)

“I would say 99.9% of the time we’ve been treated really well, like human beings, not just numbers, and that has made an enormous difference to the experience as a family.” (ID03, daughter with exomphalos)

But parents’ communication did not always go so well and parents could find this very undermining, particularly at such a stressful and worrying time. One parent (ID37) felt that

For peer review only - http://bmjopen.bmj.com/site/about/guidelines.xhtml

the doctors looking after her daughter didn't respect her wish to breastfeed, and had an attitude that parents were part of the problem rather than part of the solution, which frustrated her.

Unlike parent ID01, one mother (ID19) felt that doctors were very dismissive of the research and expert patient knowledge she had gathered from online support groups.

So yeah the surgeons need to learn how to not go 'Oh the internet' every time you suggest that someone has [laughs] mentioned this to you [laughs] because it's not just Googling symptoms and getting an answer it's asking a forum of parents who've been through exactly what you're going through. It's like sitting in a room with 500 parents whose kids have got the same experience and had the same problem who'll all have widely different experiences of it and the surgeon just going 'I'm not listening to any of you lot,' what surgeon would stand in a room of parents and do that but because its's on the internet it seems to be acceptable to go 'Tut, Facebook.' (ID19, daughter with exomphalos)

Managing information and information overload

Understanding the diagnosis and possible journey ahead can be overwhelming for parents. Parents often felt powerless, both during pregnancy and once their baby had been born. Information – about the condition and procedures but also other parent experiences – was crucial in helping parents come to terms with the diagnosis. Seeking out information played a central role, helping parents feel they were actively doing something for their baby and family. ID01's son had exomphalos. Seeking information felt like the only thing she and her husband could do. *"We were powerless to change anything other than lots of information".*

However, there were also dangers of information overload. Parents could find it hard to take on board a great deal of complex information at once, and needed time to understand and process what was happening to their baby. Parent ID22's son was diagnosed with Hirschsprung's disease. She was given an information sheet and some medical alert cards so they could identify any symptoms of things going wrong.

For peer review only - http://bmjopen.bmj.com/site/about/guidelines.xhtml

"They gave us enough information but not too much because I think they obviously realised that, you know, it's early days but they had to highlight the seriousness of it and that, know if anything if he was showing any signs of anything you're to bring him back basically." (ID22)

On the whole, parents said the information given by hospitals was not sufficient and they supplemented it with their own background reading and research. Pointing parents in the direction of trusted sources of information would be a key easy action to help them.

Encouraging parents to seek out others

Finding support from other parents who had been through similar experiences (either online or face to face) was described as a crucial factor in helping parents cope and understand more about their baby's diagnosis.

"*Find parents, my absolute number one thing."* (ID01, son with exomphalos)

"That was my number one support during that time with all these other mums who'd gone through it." (ID02, daughter with gastroschisis).

Staff can encourage and facilitate this through introducing parents to each other, or suggesting online support groups. One unit had established a popular and thriving Facebook support group for parents of babies with Hirschsprung's disease.

"It just makes it a bit real, you know, that you aren't the only one". (ID22, son with Hirschsprung's disease)

Helping parents find a role

Parents often described how "useless" or "helpless" they felt not being able to do anything for their baby while in hospital. Yet when they went home they were going to become

For peer review only

experts in their baby's care. Helping them bridge this gap was an area where staff could make a real impact.

"I think you're so wrapped up in the medical needs of your baby and hoping that they're going to be alive at the end of it that I just…hadn't anticipated that the little things would be the things that matter, it would be the 'mummy' things that had been so easy to do with my other children, they would be the things that would matter that I missed out on." (ID19, daughter with exomphalos).

In the early days, spending time at their baby's side was all many parents felt they could do. They looked for small ways they could regain a sense of control and get involved in their baby's care. While it was not always practical in critical care environments, parents appreciated helping with feeding, bathing, singing and talking to their baby where possible. After three weeks, ID02 was able to hold her daughter for the first time, "*it was like Christmas*". Supporting mothers to either breastfeed or, more often, express milk for their baby, was another way of parents bonding and being involved in their baby's care. Some parents described how they were supported and encouraged to become "experts" in aspects of their baby's daily care – changing dressings or stoma bags. One father (ID05) said he became the "dressing king"; after a rocky start. Another (ID03) became an expert at dressing her daughter's exomphalos, "*treating and dressing it was still a bit of a skill. It got easier and easier and it's a real shame. I'm now one of the finest exomphalos wrappers in [county] and it's not a skill that's really going to be used that much, is it?"* Activities like these were key to bridging the gap between hospital and home.

Preparing to go home

After weeks or months in hospital, being able to bring their baby home was a huge step for many parents. While a positive sign that their baby was on the mend, it could also be

For peer review only - http://bmjopen.bmj.com/site/about/guidelines.xhtml

daunting to leave the "safety net" of hospital and clinical expertise. Some parents described handover or normalisation programmes as very helpful in preparing them technically and emotionally for caring for their infant at home. Parents were trained in some of the skills they would need (wound management, stoma care, first aid, resuscitation) and given the opportunity to 'room in' with their baby for a few nights before going home.

"You're totally encouraged to do as much care for your baby as you can. It's your baby, they're there to support you and to medically [um] jump in if, if need be, but don't expect that they're there to feed and care for your baby because that's your job." (ID34, son with gastroschisis)

Open access policy

Accessing hospitals and specialists after their baby had been sent home could be challenging for parents, especially if home was a distance from the specialist surgical centre. When problems came up, as they often did, it was hard to know where to take their baby. So being given open or emergency access back to the team who had looked after their baby, without having to go through A&E first, was really valued by parents. ID38's son had complex needs after his surgery for oesophageal atresia and trachea-oesophageal fistula (TOF) said having open access to the ward where they can turn up with their baby and see doctors who know their baby was so important. *"I know that won't last forever but right now the open access is a Godsend because it means we get seen really quickly*."

DISCUSSION

This study adds valuable insights into the support and information needs of parents when their baby requires early surgery. These surgical journeys are often a long haul for baby and parents. Our study highlights the importance of clear and ongoing communication between parents and the often large, multi-disciplinary teams caring for their infant and points to

For peer review only - http://bmjopen.bmj.com/site/about/guidelines.xhtml

immediate actions that can improve parent experiences and outcomes. Parents value being listened to, encouraged to be a part of the care team and supported in finding a role for themselves. They need information from the professionals caring for their infant but also recommendations of good sources of information to supplement their knowledge, while being mindful of information overload. For some, encouraging them to seek support from other parents is of great value.

Previous work exploring parents' experiences of having an infant in NICU or surgery has highlighted the importance of good communication and emotional support, giving parents support at handover for surgery and pointed to the impact of scanty and infrequent information giving.[7 32] Parents experiences are not only affected by their baby's diagnosis and care, but also the ways in which services are organised to support families to navigate the systems and maintain a new 'second campus' as they look after their baby and continue their lives outside the hospital.[33]

Involving and supporting parents is increasingly recognised as an important component of providing holistic paediatric care and can help with the uncertainty of parenting an infant during critical illness. Parent experiences of NICU point to the vital role that neonatal nurses have in engaging with mothers and the importance of that relationship.[33] Aagaard[5] discussed the importance of trying to strengthen maternal competence, and suggested parent-nurse chat as a communication strategy. Our findings provide examples of the ways in which encouraging parents to be involved with infant can be achieved.

For peer review only

Others have described the role of the expert parent [33] and home monitoring programmes after cardiac surgery.[17 34] Studies have indicated that parents are not adequately prepared for discharge and are not well equipped to recognise deterioration in their child. The positive examples of being prepared for discharge and supported in becoming the primary carer offer insights into how parents might be supported to manage this transition.

Bourdieu's theoretical concepts of habitus and capital are helpful in exploring and unpacking the experiences of parents in these highly technical environments and the value they place on communication, finding their own role and seeking out other parents. They travel a journey from being newcomers in an environment (neonatal/surgical unit) where they are unfamiliar with the socialised norms. They have little capital and no idea how to 'play the game' (what Bourdieu termed the logic of practice) and negotiate the field. There is initially an unequal balance of power. Thus examples of parents feeling dismissed (or part of the problem) amplify this imbalance. Feeling included in the team caring for their infant and having their expertise (in changing dressings, feeding etc) supported and celebrated can be a powerful tool in addressing this imbalance . Over time, assisted by good communication from the health professionals and the networks of support and information provided by other parents, parents develop confidence and competency which Bourdieu might have interpreted as social and cultural capital. Their social capital is extended through the social networks they develop inside and outside the hospital environment, online and in the real world, related to their new role in caring for their infant. Their cultural capital is extended through the knowledge, expertise and skills they develop to care for their infant. By the time they take their infants home these parents are well on the way to becoming technical experts in their baby's illness and care.

For peer review only

For peer review only - http://bmjopen.bmj.com/site/about/guidelines.xhtml

STRENGTHS AND LIMITATIONS

This is a UK based study that sought to explore the experiences of parents who have an infant requiring surgery in the first year of life. Interview studies in this field are rare and our study provides rich insights into the emotional and practical impacts of these experiences. While the interviews could not cover all possible conditions, they provide insights that may be generalisable across many different surgical procedures/medical conditions. However, there are several limitations worth considering. We interviewed parents at different lengths of time after their surgery, as we sought to gain insights into the long term as well as short term impacts of these experiences. However this variation could have influenced parents' recall of events. Parents were asked to describe an intense and often protracted period of their lives in one interview, so it is perhaps inevitable that some details were omitted or compressed. There was also a variety in interviews, with some parents choosing to be interviewed alone, and others in couples. This inevitably means that some interviews are co-produced while others are not.

CONCLUSION

As babies recover, the potential for parental expertise grows. Our study highlights the vital role of information, both about the condition and about the infant's progress. Health care staff could discuss or direct parents to information sources, seek to empower parents through involving them with their infant's care where possible, and link them with other parents.

For peer review only

A Contributorship statement The study was conceived by MK. All interviews were collected by LH. Analysis was undertaken by LH and LL, with input from MK and AM. LH wrote the article, with input from the other authors.

B Competing Interests Authors declare no competing interests

C Funding: This study was funded through a National Institute for Health Research (NIHR) Professorship award to Marian Knight (NIHR-RP-011-032). The views expressed are those of the author(s) and not necessarily those of the NHS, the NIHR or the Department of Health and by the National Institute for Health Research Biomedical Research Centre, Oxford, grant BRC-1215-20008 to the Oxford University Hospitals NHS Foundation Trust and the University of Oxford.

D Data Sharing statement Participants were invited to review their transcript and mark any sections that they did not want used before transferring copyright to the University of Oxford for use in research, teaching, publications and broadcasting. These carefully anonymised transcripts form part of a University of Oxford archive which is available to other bona fide research teams for secondary analysis. All authors had access to the all of the data in the study and take responsibility for the integrity of the data and the accuracy of the data analysis.

For peer review only

REFERENCES

1. Allin BSR, Tse WHW, Marven S, et al. Challenges of Improving the Evidence Base in Smaller Surgical Specialties, as Highlighted by a Systematic Review of Gastroschisis Management. *Plos One* 2015;10(1) doi: ARTN e0116908
10.1371/journal.pone.0116908
2. Allin B, Ross A, Marven S, et al. Development of a core outcome set for use in determining the overall success of gastroschisis treatment. *Trials* 2016;17 doi: ARTN 360
10.1186/s13063-016-1453-7
3. Lam HS, Sit T, Chau CL, et al. Attitudes of Parents and Health Care Workers to Major Surgery for High-Risk Preterm Infants. *The Journal of Pediatrics* 2016;177(Supplement C):78-83.e3. doi: https://doi.org/10.1016/j.jpeds.2016.06.071
4. Tam PKH, Chung PHY, St Peter SD, et al. Advances in paediatric gastroenterology. *Lancet* 2017;390(10099):1072-82.
5. Aagaard H, Hall EOC. Mothers' Experiences of Having a Preterm Infant in the Neonatal Care Unit: A Meta-Synthesis. *Journal of Pediatric Nursing* 2008;23(3):e26-e36. doi: https://doi.org/10.1016/j.pedn.2007.02.003
6. Wei H, Roscigno CI, Hanson CC, et al. Families of children with congenital heart disease: A literature review. *Heart Lung* 2015;44(6):494-511. doi: 10.1016/j.hrtlng.2015.08.005
7. Wigert H, Dellenmark MB, Bry K. Strengths and weaknesses of parent-staff communication in the NICU: a survey assessment. *Bmc Pediatr* 2013;13 doi: Artn 71
10.1186/1471-2431-13-71
8. Jordan B, Franich-Ray C, Albert N, et al. Early mother-infant relationships after cardiac surgery in infancy. *Arch Dis Child* 2014;99(7):641-5. doi: 10.1136/archdischild-2012-303488
9. Woolf-King SE, Anger A, Arnold EA, et al. Mental Health Among Parents of Children With Critical Congenital Heart Defects: A Systematic Review. *J Am Heart Assoc* 2017;6(2) doi: ARTN e004862
10.1161/JAHA.116.004862
10. Aite L, Bevilacqua F, Zaccara A, et al. Seeing Their Children in Pain: Symptoms of Posttraumatic Stress Disorder in Mothers of Children with an Anomaly Requiring Surgery at Birth. *Am J Perinat* 2016;33(8):770-75. doi: 10.1055/s-0036-1572543
11. Le Gouez M, Alvarez L, Rousseau V, et al. Posttraumatic Stress Reactions in Parents of Children Esophageal Atresia. *Plos One* 2016;11(3) doi: ARTN e0150760
10.1371/journal.pone.0150760
12. Kaasen A, Helbig A, Malt UF, et al. Acute maternal social dysfunction, health perception and psychological distress after ultrasonographic detection of a fetal structural anomaly. *BJOG* 2010;117(9):1127-38. doi: 10.1111/j.1471-0528.2010.02622.x
13. Connor JA, Kline NE, Mott S, et al. The Meaning of Cost for Families of Children With Congenital Heart Disease. *J Pediatr Health Car* 2010;24(5):318-25. doi: 10.1016/j.pedhc.2009.09.002
14. Froh EB, Deatrick JA, Curley MAQ, et al. Making Meaning of Pumping for Mothers of Infants With Congenital Diaphragmatic Hernia. *Jognn-J Obst Gyn Neo* 2015;44(3):439-49. doi: 10.1111/1552-6909.12564
15. Ainsworth MDB, M; Waters, E. . Patterns of attachment: a psychological study of the strange situation. New Jersey: Erlbaum 1978.

For peer review only - http://bmjopen.bmj.com/site/about/guidelines.xhtml

16. Bowlby J. The nature of the child's tie to the mother. *International Journal of Psychoanalysis* 1958;38(9):350-72.
17. Tregay J, Brown KL, Crowe S, et al. Signs of deterioration in infants discharged home following congenital heart surgery in the first year of life: a qualitative study. *Arch Dis Child* 2016;101(10):902-8. doi: 10.1136/archdischild-2014-308092
18. Lupton D, Fenwick J. 'They've forgotten that I'm the mum': constructing and practising motherhood in special care nurseries. *Soc Sci Med* 2001;53(8):1011-21. doi: Doi 10.1016/S0277-9536(00)00396-8
19. Wilkinson D. Death or Disability? The 'Carmentis Machine' and decision-making for critically ill children. Oxford: Oxford University Press 2013.
20. Kubota A, Yamakawa S, Yamamoto E, et al. Major neonatal surgery: psychosocial consequence of the patient and mothers. *J Pediatr Surg* 2016;51(3):364-67. doi: 10.1016/j.jpedsurg.2015.09.017
21. Laing S, McMahon C, Ungerer J, et al. Mother–child interaction and child developmental capacities in toddlers with major birth defects requiring newborn surgery. *Early Human Development* 2010;86(12):793-800. doi: https://doi.org/10.1016/j.earlhumdev.2010.08.025
22. Kratochvil MS, Robertson CMT, Kyle JM. Parents' View of Parent-Child Relationship Eight Years After Neonatal Intensive Care. *Social Work in Health Care* 1992;16(1):95-118. doi: 10.1300/J010v16n01_06
23. Huppatz K. Pierre Bourdieu: Health Lifestyles, the Family and Social Class. In: Collyer F, ed. Palgrave Handbook of Social Theory in Health, Illness and Medicine. Basingstoke: Palgrave Macmillan 2015:370-88.
24. Webb J, Schirato, T, Danaher, G, . Understanding Bourdieu. London: Sage 2002.
25. Locock L, Boylan, A-M., Snow, R., Staniszweska, S. The power of symbolic capital in patient and public involvement in health research. *Health Expectations* 2016 doi: DOI: 10.1111/hex.12519
26. Bourdieu P. What makes a social class? On the theoretical and practical existence of groups. *Berkeley J Sociol* 1987;32:1-17.
27. Bourdieu P. Distinction: A Critique of the Judgement of Taste. London: Routledge 1984.
28. Green JT, N. Qualitative Methods in Health Research. London: Sage 2004.
29. Glaser BS, Strauss A. The Discovery of Grounded Theory. New York: Adine Publishing 1967.
30. Green JT, Thorogood N. Qualitative Methods for Health Research. London: Sage 2004.
31. Pope C, Ziebland S, Mays N. Qualitative research in health care: Analysing qualitative data. *BMJ: British Medical Journal* 2000;320(7227):114.
32. Wei H, Roscigno CI, Swanson KM, et al. Parents' experiences of having a child undergoing congenital heart surgery: An emotional rollercoaster from shocking to blessing. *Heart & Lung: The Journal of Acute and Critical Care* 2016;45(2):154-60. doi: https://doi.org/10.1016/j.hrtlng.2015.12.007
33. Kosta L, Harms L, Franich-Ray C, et al. Parental experiences of their infant's hospitalization for cardiac surgery. *Child Care Hlth Dev* 2015;41(6):1057-65. doi: 10.1111/cch.12230
34. Gaskin KL, Barron DJ, Daniels A. Parents' preparedness for their infants' discharge following first-stage cardiac surgery: development of a parental early warning tool. *Cardiol Young* 2016;26(7):1414-24. doi: 10.1017/S1047951116001062

For peer review only

Table of Participants

Table 1 Participants

Condition	Identifier	Parent Mother/Father	Sex of child	Child's age at interview	Intensive or high dependency care admission
Exomphalos*	01	M	m	18 months	Yes
	02	M	f	6 years	Yes
	03	M	f	8 years	Yes
	04/05	M&F	f	8 years	Yes
	12/13	M&F	m	25 years	No
	16/17	M&F	m	13 years	Yes
	19	M	f	16 months	Yes
	41/42	M&F	m	Died age 7 months	Yes
Necrotising Enterocolitis	08	M	m	9 months	Yes
	09	M	m	11 months	Yes
	23	M	m	6 months	Yes
Hernia	26/27	M&F	f	9 months	No
Congenital Diaphragmatic Hernia*	10/11	M&F	m	9 months	Yes
	15	M	m	5 years	Yes
	24	M	m	9 years	Yes
Hirschprung's Disease*	18	M	f	1 year	No
	20/21	M&F	m	4 months	Yes
	22	M	m	16 months	No
	28/29	M&F	m	19 months	No
	39/40	M&F	m	5 years old	No
	43/44	M&F	m	5 years old	No
Gastroschisis*	30	M	m	7 ½ months	Yes
	31	M	m	4 years	Yes
	32	M	f	3 years	Yes
	33/34	M&F	m	3 months	Yes
	36	M	f	19 months	Yes
	37	M	f	6 years	Yes
Short Bowel	35	M	f	7 years	Yes
Jejunal atresia*	25	M	f	4 months	Yes

Oesophageal Atresia with trachea-oesophageal fistula*	38		m	19 months	Yes
Undiagnosed	14		m	6 months	
*Congenital diagnosis					

For peer review only

COREQ (COnsolidated criteria for REporting Qualitative research) Checklist

A checklist of items that should be included in reports of qualitative research. You must report the page number in your manuscript where you consider each of the items listed in this checklist. If you have not included this information, either revise your manuscript accordingly before submitting or note N/A.

Topic	Item No.	Guide Questions/Description	Reported on Page No.
**Domain 1: Research team and reflexivity**			
Personal characteristics			
Interviewer/facilitator	1	Which author/s conducted the interview or focus group?	p.8
Credentials	2	What were the researcher's credentials? E.g. PhD, MD	p.1 & 8
Occupation	3	What was their occupation at the time of the study?	p.1
Gender	4	Was the researcher male or female?	p.1
Experience and training	5	What experience or training did the researcher have?	p.1
Relationship with participants			
Relationship established	6	Was a relationship established prior to study commencement?	NO
Participant knowledge of the interviewer	7	What did the participants know about the researcher? e.g. personal goals, reasons for doing the research	p.8
Interviewer characteristics	8	What characteristics were reported about the inter viewer/facilitator? e.g. Bias, assumptions, reasons and interests in the research topic	p.8
**Domain 2: Study design**			
Theoretical framework			
Methodological orientation and Theory	9	What methodological orientation was stated to underpin the study? e.g. grounded theory, discourse analysis, ethnography, phenomenology, content analysis	p.6 & 9
Participant selection			
Sampling	10	How were participants selected? e.g. purposive, convenience, consecutive, snowball	p.8
Method of approach	11	How were participants approached? e.g. face-to-face, telephone, mail, email	p.8
Sample size	12	How many participants were in the study?	p.7
Non-participation	13	How many people refused to participate or dropped out? Reasons?	p.7
Setting			
Setting of data collection	14	Where was the data collected? e.g. home, clinic, workplace	p.8
Presence of non-participants	15	Was anyone else present besides the participants and researchers?	No
Description of sample	16	What are the important characteristics of the sample? e.g. demographic data, date	Table 1
Data collection			
Interview guide	17	Were questions, prompts, guides provided by the authors? Was it pilot tested?	No, but can be
Repeat interviews	18	Were repeat inter views carried out? If yes, how many?	No
Audio/visual recording	19	Did the research use audio or visual recording to collect the data?	p.8
Field notes	20	Were field notes made during and/or after the interview or focus group?	Yes
Duration	21	What was the duration of the inter views or focus group?	p.9
Data saturation	22	Was data saturation discussed?	p.9
Transcripts returned	23	Were transcripts returned to participants for comment and/or	.p.8

For peer review only

Topic	Item No.	Guide Questions/Description	Reported on Page No.
		correction?	
**Domain 3: analysis and findings**			
Data analysis			
Number of data coders	24	How many data coders coded the data?	p.9
Description of the coding tree	25	Did authors provide a description of the coding tree?	No
Derivation of themes	26	Were themes identified in advance or derived from the data?	p.9
Software	27	What software, if applicable, was used to manage the data?	p.9
Participant checking	28	Did participants provide feedback on the findings?	N/A
Reporting			
Quotations presented	29	Were participant quotations presented to illustrate the themes/findings? Was each quotation identified? e.g. participant number	P.10-17
Data and findings consistent	30	Was there consistency between the data presented and the findings?	Yes
Clarity of major themes	31	Were major themes clearly presented in the findings?	Yes
Clarity of minor themes	32	Is there a description of diverse cases or discussion of minor themes?	Yes

Developed from: Tong A, Sainsbury P, Craig J. Consolidated criteria for reporting qualitative research (COREQ): a 32-item checklist for interviews and focus groups. *International Journal for Quality in Health Care*. 2007. Volume 19, Number 6: pp. 349 – 357

Once you have completed this checklist, please save a copy and upload it as part of your submission. DO NOT include this checklist as part of the main manuscript document. It must be uploaded as a separate file.

For peer review only

BMJ Open

What can make things better for parents when babies need abdominal surgery in their first year of life? A qualitative interview study in the United Kingdom

Journal:	*BMJ Open*
Manuscript ID	bmjopen-2017-020921.R2
Article Type:	Research
Date Submitted by the Author:	29-Mar-2018
Complete List of Authors:	Hinton, Lisa; University of Oxford, Health Experiences Research Group, Nuffield Department of Primary Care Health Sciencs<br>Locock, Louise; University of Aberdeen, Health Service Research<br>Long, Anna-May; University of Oxford, National Perinatal Epidemiology Unit<br>Knight, Marian; National Perinatal Epidemiology Unit
**Primary Subject Heading**:	Paediatrics
Secondary Subject Heading:	Surgery, Sociology, Qualitative research
Keywords:	Paediatric gastroenterology < GASTROENTEROLOGY, Paediatric intensive & critical care < INTENSIVE & CRITICAL CARE, QUALITATIVE RESEARCH, Pancreatic surgery < SURGERY

SCHOLARONE™
Manuscripts

For peer review only

What can make things better for parents when babies need abdominal surgery in their first year of life? A qualitative interview study in the United Kingdom

Hinton L, Locock L, Long A-M, Knight M.

Author information

Lisa Hinton, DPhil, Nuffield Department of Primary Care Health Sciences, University of Oxford, Oxford. OX2 6GG, lisa.hinton@phc.ox.ac.uk, +44 (0)1865 289304

Louise Locock, Professor of Health Services Research, University of Aberdeen, Scotland AB25 2ZD

Marian Knight, NIHR Professor of Maternal and Child Population Health, National Perinatal Epidemiology Unit, University of Oxford OX3 7LF

Anna-May Long, Clinical Research Fellow in Paediatric Surgery

Word count [4713]

For peer review only

For peer review only

ABSTRACT

(i) Objectives

To understand the experiences of parents of infants who required surgery early in life. To identify messages and training needs for the extended clinical teams caring for these families – including paediatric surgeons, neonatologists, nurses, obstetricians, midwives and sonographers.

(ii) Setting

United Kingdom wide interview study, including England, Wales and Scotland.

(iii) Participants

In-depth interviews were conducted with 44 parents who had a baby who underwent early abdominal surgery. Conditions included those diagnosed antenatally (e.g. exomphalos, gastroschisis, congenital diaphragmatic hernia) or which were detected postnatally (e.g. Hirschsprung's disease, necrotising enterocolitis). Interviews were video and audio recorded and analysed using a modified grounded-theory approach.

(iv) **Results**

While some parents reported experiencing excellent communication and felt they were listened to and involved by the care team, this was not always the case. Dealing with large, complex medical and surgical teams could result in conflicting messages, uncertainty and distress. Parents wanted information but also described being overwhelmed and wanting to distance themselves to maintain hope. Information and support from other parents in hospital and online groups was highly valued. Of particular concern was support when going home and caring for their baby after discharge; an open access policy for readmission offered a helpful safety net.

(v) Conclusions

For peer review only

Listening to the experience of parents provides rich data to enhance clinical understandings on how to improve information and communication with parents, and ameliorate the deep and lasting distress and anxiety that some parents feel when their infants face early surgery. We suggest that the writings of Bourdieu could have resonance in interpreting the experiences of parents as they enter the world of highly-technical neonatal medicine and surgery and the knowledge of the professionals who work in these environments.

Article Summary

Strengths and Limitations

- This is a UK based study that sought to explore the experiences of parents who have an infant requiring abdominal surgery in the first year of life.
- Interview studies in this field are rare and our study provides rich insights into the emotional and practical impacts of these experiences.
- While the interviews could not cover all possible conditions, they provide insights that may be generalisable across many different surgical procedures/medical conditions.
- Interviews were conducted at varying distances from the event, so recall was varied. Parents described an intense and often protracted period of their lives, so some details may have been omitted or compressed in their accounts.

INTRODUCTION

The experiences of parents of infants who undergo abdominal surgery in the first year of life are rarely recorded. Even after discharge from hospital, these infants may require multiple outpatient visits to different specialist clinics and their ongoing care

For peer review only

requirements can have significant effects on the quality of life of parents and existing children, as well as economic effects through lost work days.

Many of these infants will require surgery because of a gastrointestinal congenital anomaly, such as exomphalos, gastroschisis or Hirschsprung's disease. These conditions are under-researched[1], understanding of them is poor and interventions are rarely based on robust evidence. Routine sources of information are limited.[2] Outcomes for infants with rare anomalies are improving[3 4] and so the need for rigorous research into surgical, clinical and long-term outcomes is paramount.[2] Alongside this, understanding the impact of these diagnoses and surgical treatments on parents and families is central to their long-term recovery.

This study was conducted as an adjunct to the British Association of Paediatric Surgeons Congenital Anomaly Surveillance System (BAPS-CASS) and was focused on parents' experiences of abdominal surgery. There is little research on parent experiences of conditions that require early abdominal surgery. However, we can supplement this limited evidence base by drawing on research into parent experiences of congenital heart disease and surgery and more generally research about parents' experiences of neonatal intensive care units. The literature here points to the practical and psychological impact on families [5 6] and the importance of good communication between parents and staff.[7] Parents of children with congenital heart disease are at high risk for mental health morbidity, experiencing stress, depression, anxiety and traumatic stress responses [8-12] and the cost implications are profound.[13] Parents experience difficulties with bonding and breastfeeding while their infants are in hospital [8 14 15 16]and their infants' complex needs after discharge.[17]

For peer review only

Mothers of infants in neonatal intensive care face emotional challenges as they seek to find a role for themselves in medically complex environments with perilously ill infants.[18] Wilkinson[19] and others have explored the contentious ethical issues facing those who care for critically ill infants and the extent to which parents should be involved in decision-making. Little is known about the relationship between quality of mother-child interaction and child development after major neonatal surgery or intensive care, but the parental mental health and familial environment are thought to be vital to the recovery and mental well-being of the infant.[20-22]

Theoretical framework

One approach to thinking about these experiences could be to draw on the writings of the French sociologist Pierre Bourdieu whose work focused on examining experiences of social class and the production of knowledge and power [23 24]. One of his key concepts was the notion of 'habitus', whereby individuals share their history and environment with others who have similar positions, producing a 'stylistic affinity' and where 'social identity is defined and asserted through difference'. Habitus is the way one unconsciously acts, interacts and behaves within the social world in a 'taken for granted' manner, according to socialised norms, traditions and unwritten rules of particular groups[25]. Bourdieu identifies various forms of 'capital', economic, social and cultural resources that establish social standing within a particular habitus or social setting. Power is derived from configurations of these types of capital[26] within a given field (in this case neonatal care and surgery). Although his writings focused on class lifestyles and social identity, these ideas could have resonance in interpreting the experiences of parents as they enter the world of highly-technical

For peer review only - http://bmjopen.bmj.com/site/about/guidelines.xhtml

neonatal medicine and surgery and the knowledge of the professionals who work in these environments[27].

The aim of this qualitative study was to: explore parents' experiences and perspectives of having a baby who needs early abdominal surgery; identify the questions and problems that matter to parents during and after their pregnancy and infant's surgery; and identify the long-term impact on parents and families. In this article, we report on the period immediately before and after surgery, and suggest actions that can be implemented to rapidly improve parents' experiences. Findings on the longer-term impacts of these experiences will be reported elsewhere.

METHODS

We conducted interviews with 44 parents, all of whom had an infant who needed abdominal surgery in the first year of life. Two parents did not return their copyright form so their data is not included in our analysis.

Participants

Parents were recruited from England, Scotland and Wales. We interviewed parents whose infant had required surgery in their first year of life. We were keen to capture long term perspectives on the experiences, as well as recent ones. So we sought parents whose infant or child was now a wide variety of differing ages. Therefore participants included those for whom the experience was very recent through to parents whose son was 25 years old. Most parents' infants were still living, but we interviewed the parents of one boy who died aged seven months. We interviewed some mothers individually and eleven couples (mother and father). Conditions that

For peer review only

their infants had been diagnosed with included exomphalos, gastroschisis, congenital diaphragmatic hernia, Hirschsprung's disease, inguinal hernia, atresia (jejunal and oesophageal) and necrotising enterocolitis (see Table 1). Some conditions were diagnosed antenatally, others developed after their baby was born. Some conditions were congenital, others developed as a result of premature birth or spontaneously (see Table 1). All infants had their surgery in the first year of life, although some went on to have multiple operations. Several spent extended periods of time in neonatal intensive care units (see Table 1).

Interviews

Parents were interviewed either individually or in pairs, depending on preference. We interviewed eleven fathers, but none chose to be interviewed on their own. Recruitment was through support groups, paediatric surgeons, neonatal nurses, other specialists and word of mouth. We aimed for a maximum variation sample and continued interviews until thematic saturation was reached. All interviews were conducted by LH, a social scientist, in participants' own homes, or in a venue of their choice using a semi-structured narrative approach[28]. The interviews were in two parts, beginning with an invitation to offer an unstructured narrative, prompted by an open-ended question at the start of the interview. This was followed up by a semi-structured component of the interview with prompts to follow up issues raised in the narrative and to explore themes suggested by the literature and the advisory panel and a group of parent advisors. The interview schedule was developed by co-authors in consultation with the study advisory panel and parent advisors. All interviews were audio recorded, and where consent was given video recorded to facilitate dissemination on the patient experience website, healthtalk.org. Parents

For peer review only - http://bmjopen.bmj.com/site/about/guidelines.xhtml

were offered a two-stage consent and copyright approval process. They signed a consent form before the interview started and were subsequently sent a copy of their transcript to approve before signing a copyright form agreeing to their data being included in the analysis and excerpts from their interviews included in dissemination, including peer review articles, education materials and on the healthtalk.org website. Those who wanted to remain anonymous could choose to have only audio or written-only excerpts of their interviews included, and a pseudonym. Interviews lasted from between 75 minutes and 3 and a half hours. All interviews were fully transcribed. We undertook analysis of the transcribed interviews using an interpretive approach to thematic analysis [29 30]. Interview transcripts, not videos, were coded by LH with support from NVIVO analysis software, using a framework which was developed iteratively and reflected both anticipated and emergent themes. Coding reports were then analysed separately by LH and LL using a modified grounded theory approach, incorporating constant comparison and exploration of deviant cases, allowing the data to be grouped into themes and all cases to be examined to ensure all the manifestations of each theme were accounted for and compared[31] and we were satisfied we had reached data saturation with no new constructs emerging [30]. Any differences in interpretation were then discussed. MK and AML reviewed summarised findings and contributed a further clinical layer of analysis.

Patient and Public Involvement

A parent advisory panel was established at the inception of the study and met annually. Parents with lived experiences of having an infant who required neonatal surgery were involved in the face to face meetings or via email. Parents contributed to the design of the sample and interview schedule, helped with recruitment and

For peer review only

provided feedback on initial analysis and the final documents published on the Healthtalk.org website. Parents were invited to a launch event for the website.

Ethics Committee Approval: Ethics committee approval was given for this study by the Berkshire Ethics Committee, 09/H0505/66. All participants gave informed consent before taking part and have given written consent to their interview data being included in publications.

RESULTS

Some parents discovered there was a problem with their baby during antenatal scans, others not until after their baby had been born. Regardless of when the diagnosis came, parents of a baby who needs abdominal surgery faced many challenges over the ensuing months and years.

The care pathways for these infants were often long and complex. Setbacks were common, and at no point could clinical staff give parents any guarantees. The practical and emotional uncertainties were, therefore, huge. Living with uncertainty and worry was something parents had to get used to, at every stage. Many parents described a lasting distress and anxiety.

"It was always a waiting game and tricky because you want to know the answer, 'When is my baby going to come home?'" (ID02, mother, daughter with gastroschisis)

"*We just don't know what road he is going to take."* (ID20, mother, son with Hirschsprung's disease)

For peer review only

"My anxiety levels have been awful really it's been hard for everybody because I've been up and down emotionally". (ID23, mother, son with NEC)

Large and complex teams of staff across many different specialities were involved in looking after these infants over weeks, months and years. Our interviews highlighted some key steps that could improve the experience for parents. These do not so much focus on the surgery itself, but on the care and support parents are given before and after.

i) Communication
ii) Managing information and information overload
iii) Encouraging parents to seek out others (online, hospital groups)
iv) Help in finding a role
v) Preparing for going home
vi) Open access policy

Communication

Communication with the various health professionals looking after their baby was central to parents' experiences. These professionals spanned many disciplines – neonatologists, surgeons, but also paediatricians, intensive care nurses, specialist stoma nurses and other allied health professionals further along, such as physiotherapists or nutritionists.

For peer review only - http://bmjopen.bmj.com/site/about/guidelines.xhtml

The babies were often very sick with a rare condition and receiving complex medical and surgical treatments. Parents often felt isolated and struggled to find information about their baby's diagnosis and condition. Communication about their baby's progress and the treatment plan was therefore very important.

"If you're in the dark that's when people would worry [….] as long as you, you know, either good or bad, what was going on and a lot of the things you ask them they don't know the answer because it's a time will tell kind of answer, but so long as someone tells you the time will tell sort of answer you've got an answer." (ID25, mother, daughter with jejunal atresia)

There were often extended periods of uncertainty as doctors were not able to answer parents' questions. While these periods were a challenge to parents, there were examples of good, clear communication which really helped at a stressful, frightening time.

i) Being kept up to date

Being kept up to date with their baby's care was important to parents, especially when plans changed. They valued the opportunity to ask questions repeatedly. ID25 said she was constantly asking questions but staff were very good at answering them all and reassuring her.

"[I] probably annoyed the hell out of them, but wanted to know, OK, when he does this what is his next step, what has progressed to, how long will he be doing that and how long will it take and things." (ID15, mother, son with CDH)

The nurses were good as well at feeding back what the doctors had said and also if you had any questions I felt comfortable saying look can you ask about this and let me know what they say, so. (ID30, mother, son with gastroschisis)

For peer review only - http://bmjopen.bmj.com/site/about/guidelines.xhtml

ii) Communicating well

Although health professionals often had to deliver bad news, there were many examples of good practice in managing this well. These interactions were characterised by giving parents information in a clear, accessible manner, sometimes supported by drawing diagrams, but without patronising or 'dumbing down'. Parents appreciated doctors and nurses taking time to answer their questions, not making them feel that any question was a silly one.

M*: And went through them with the consultant and actually again that was another thing that he very patiently sat and went through every single one of those questions, however stupid they were, you know.*

F: *How many times he'd heard them, no doubt.*

M: *Yeah and it didn't.*

F: *He wasn't fazed, he wasn't awkward he was just 'Okay, let's do it'.*
(ID 43/44, mother and father, son with Hirschsprung's disease)

"I quite like things in a lot of detail so that when doctors are talking to you sometimes they can do stupid talk because they don't know how much you know medically, but I like to hear all of it and I don't want broken down terms, I want medical terms because then I can go and look up what the medical terms are, see what it's all about." (ID25, mother, daughter with jejunal atresia)

iii) Conveying expertise

When infants were in hospital for long periods, relationships of mutual respect between parents and healthcare professionals were hugely valued. As one mother explained, "you are handing over the most precious thing to this person who is going to put them under anaesthetic and disappear into an operating theatre with them and you hope their gonna come out the other side. So you have to have that level of assurance." She and her husband were reassured by their surgeon's "confidence not arrogance". Others talked about the importance of trust and feeling as though their

For peer review only

baby was in expert hands, cared for by clinicians who were knowledgeable about their condition.

"They've got this under control, they really know what they are doing…… In a way it's silly thinking that now because of course they know what they're doing these are highly trained professionals but in that moment that's what you want to hear cos you just want to, you're handing over your child so you want someone to be right on it don't you and he was." (ID26, father, daughter with iguinal hernia)

iv) Being listened to and feeling part of the team

While parents recognised that they relied on doctors' expertise and judgement in planning their baby's care, they really valued feeling involved in caring for their baby where they could – "part of it, part of that team".

"They've been very understanding and patient and I think recognising as well our role as parents and, that we, you know, have opinions and experiences, and not devaluing what we've learnt from other places as well." (ID01, mother, son with exomphalos)

"I would say 99.9% of the time we've been treated really well, like human beings, not just numbers, and that has made an enormous difference to the experience as a family." (ID03, mother, daughter with exomphalos)

But communication did not always go so well and parents could find this very undermining, particularly at such a stressful and worrying time. One parent (ID37) felt that the doctors looking after her daughter didn't respect her wish to breastfeed, and had an attitude that parents were part of the problem rather than part of the solution, which frustrated her.

Unlike parent ID01, one mother (ID19) felt that doctors were very dismissive of the research and expert patient knowledge she had gathered from online support groups.

So yeah the surgeons need to learn how to not go 'Oh the internet' every time you suggest that someone has [laughs] mentioned this to you [laughs] because it's not just Googling symptoms and getting an answer it's asking a forum of parents who've

For peer review only

been through exactly what you're going through. It's like sitting in a room with 500 parents whose kids have got the same experience and had the same problem who'll all have widely different experiences of it and the surgeon just going 'I'm not listening to any of you lot,' what surgeon would stand in a room of parents and do that but because its's on the internet it seems to be acceptable to go 'Tut, Facebook.' (ID19, mother, daughter with exomphalos)

Managing information and information overload

Understanding the diagnosis and possible journey ahead can be overwhelming for parents. Parents often felt powerless, both during pregnancy and once their baby had been born. Information – about the condition and procedures but also other parent experiences – was crucial in helping parents come to terms with the diagnosis. Seeking out information played a central role, helping parents feel they were actively doing something for their baby and family. ID01's son had exomphalos. Seeking information felt like the only thing she and her husband could do. *"We were powerless to change anything other than lots of information".*

However, there were also dangers of information overload. Parents could find it hard to take on board a great deal of complex information at once, and needed time to understand and process what was happening to their baby. ID22's son was diagnosed with Hirschsprung's disease. She was given an information sheet and some medical alert cards so they could identify any symptoms of things going wrong.

"They gave us enough information but not too much because I think they obviously realised that, you know, it's early days but they had to highlight the seriousness of it and that, know if anything if he was showing any signs of anything you're to bring him back basically." (ID22, mother, son with Hirschsrpung's disease)

On the whole, parents said the information given by hospitals was not sufficient and they supplemented it with their own background reading and research. Pointing

For peer review only

parents in the direction of trusted sources of information would be a key easy action to help them.

Encouraging parents to seek out others

Finding support from other parents who had been through similar experiences (either online or face to face) was described as a crucial factor in helping parents cope and understand more about their baby's diagnosis.

"*Find parents, my absolute number one thing.*" (ID01, mother, son with exomphalos)

"That was my number one support during that time with all these other mums who'd gone through it." (ID02, mother, daughter with gastroschisis).

Staff can encourage and facilitate this through introducing parents to each other, or suggesting online support groups. One unit had established a popular and thriving Facebook support group for parents of babies with Hirschsprung's disease.

"It just makes it a bit real, you know, that you aren't the only one". (ID22, mother, son with Hirschsprung's disease)

Helping parents find a role

Parents often described how "useless" or "helpless" they felt not being able to do anything for their baby while in hospital. Yet when they went home they were going to become experts in their baby's care. Helping them bridge this gap was an area where staff could make a real impact.

"I think you're so wrapped up in the medical needs of your baby and hoping that they're going to be alive at the end of it that I just…hadn't anticipated that the little things would be the things that matter, it would be the 'mummy' things that had been so easy to do with my other children, they would be the things that would matter that I missed out on." (ID19, mother, daughter with exomphalos).

For peer review only - http://bmjopen.bmj.com/site/about/guidelines.xhtml

In the early days, spending time at their baby's side was all many parents felt they could do. They looked for small ways they could regain a sense of control and get involved in their baby's care. While it was not always practical in critical care environments, parents appreciated helping with feeding, bathing, singing and talking to their baby where possible. After three weeks, ID02 was able to hold her daughter for the first time, "*it was like Christmas*". Supporting mothers to either breastfeed or, more often, express milk for their baby, was another way of parents bonding and being involved in their baby's care. Some parents described how they were supported and encouraged to become "experts" in aspects of their baby's daily care – changing dressings or stoma bags. One father (ID05) said he became the "dressing king"; after a rocky start. Another (ID03) became an expert at dressing her daughter's exomphalos, "*treating and dressing it was still a bit of a skill. It got easier and easier and it's a real shame. I'm now one of the finest exomphalos wrappers in [county] and it's not a skill that's really going to be used that much, is it?"* Activities like these were key to bridging the gap between hospital and home.

Preparing to go home

After weeks or months in hospital, being able to bring their baby home was a huge step for many parents. While a positive sign that their baby was on the mend, it could also be daunting to leave the "safety net" of hospital and clinical expertise. Some parents described handover or normalisation programmes as very helpful in preparing them technically and emotionally for caring for their infant at home. Parents were trained in some of the skills they would need (wound management, stoma care, first aid, resuscitation) and given the opportunity to 'room in' with their baby for a few nights before going home.

For peer review only - http://bmjopen.bmj.com/site/about/guidelines.xhtml

"You're totally encouraged to do as much care for your baby as you can. It's your baby, they're there to support you and to medically [um] jump in if, if need be, but don't expect that they're there to feed and care for your baby because that's your job." (ID34, mother, son with gastroschisis)

Open access policy

Accessing hospitals and specialists after their baby had been sent home could be challenging for parents, especially if home was a distance from the specialist surgical centre. When problems came up, as they often did, it was hard to know where to take their baby. So being given open or emergency access back to the team who had looked after their baby, without having to go through A&E first, was really valued by parents. ID38's son had complex needs after his surgery for oesophageal atresia and trachea-oesophageal fistula (TOF). She said having open access to the ward where they can turn up with their baby and see doctors who know their baby was so important. *"I know that won't last forever but right now the open access is a Godsend because it means we get seen really quickly."*

DISCUSSION

This study adds valuable insights into the support and information needs of parents when their baby requires early abdominal surgery. These surgical journeys are often a long haul for baby and parents. Our study highlights the importance of clear and ongoing communication between parents and the often large, multi-disciplinary teams caring for their infant and points to immediate actions that can improve parent experiences and outcomes. Parents value being listened to, encouraged to be a part of the care team and supported in finding a role for themselves. They need information from the professionals caring for their infant but also recommendations of good sources of information to supplement their knowledge, while being mindful of

For peer review only - http://bmjopen.bmj.com/site/about/guidelines.xhtml

information overload. For some, encouraging them to seek support from other parents is of great value.

Previous work exploring parents' experiences of having an infant in NICU or surgery has highlighted the importance of good communication and emotional support, giving parents support at handover for surgery and pointed to the impact of scanty and infrequent information giving [7 32] . Parents experiences are not only affected by their baby's diagnosis and care, but also the ways in which services are organised to support families to navigate the systems and maintain a new 'second campus' as they look after their baby and continue their lives outside the hospital.[33]

Involving and supporting parents is increasingly recognised as an important component of providing holistic paediatric care and can help with the uncertainty of parenting an infant during critical illness. Parent experiences of NICU point to the vital role that neonatal nurses have in engaging with, in particular, mothers and the importance of that relationship.[33] Aagaard[5] discussed the importance of trying to strengthen maternal competence, and suggested parent-nurse chat as a communication strategy. Our findings provide examples of the ways in which encouraging parents to be involved with their infant can be achieved.

Others have described the role of the expert parent [33] and home monitoring programmes after cardiac surgery[17 34] . Studies have indicated that parents are not adequately prepared for discharge and are not well equipped to recognise deterioration in their child. The positive examples of being prepared for discharge

For peer review only - http://bmjopen.bmj.com/site/about/guidelines.xhtml

and supported in becoming the primary carer offer insights into how parents might be supported to manage this transition.

Bourdieu's theoretical concepts of habitus and capital are helpful in exploring and unpacking the experiences of parents in these highly technical environments and the value they place on communication, finding their own role and seeking out other parents. They travel a journey from being newcomers in an environment (neonatal/surgical unit) where they are unfamiliar with the socialised norms. They have little capital and no idea how to 'play the game' (what Bourdieu termed the logic of practice) and negotiate the field. There is initially an unequal balance of power. Thus, examples of parents feeling dismissed (or part of the problem) amplify this imbalance. Feeling included in the team caring for their infant and having their expertise (in changing dressings, feeding etc.) supported and celebrated can be a powerful tool in addressing this imbalance. Over time, assisted by good communication from the health professionals and the networks of support and information provided by other parents, parents develop confidence and competency - which Bourdieu might have interpreted as social and cultural capital. Their social capital is extended through the social networks they develop inside and outside the hospital environment, online and in the real world, relating to their new role in caring for their infant. Their cultural capital is extended through the knowledge, expertise and skills they develop to care for their infant. By the time they take their infants home these parents are well on the way to becoming technical experts in their baby's illness and care.

For peer review only - http://bmjopen.bmj.com/site/about/guidelines.xhtml

STRENGTHS AND LIMITATIONS

This is a UK based study that sought to explore the experiences of parents who have an infant requiring abdominal surgery in the first year of life. Interview studies in this field are rare and our study provides rich insights into the emotional and practical impacts of these experiences. While the interviews could not cover all possible conditions, they provide insights that may be generalisable across many different surgical procedures/medical conditions. However, there are several limitations worth considering. We interviewed parents at different lengths of time after their surgery, as we sought to gain insights into the long term as well as short term impacts of these experiences. However, this variation could have influenced parents' recall of events. Parents were asked to describe an intense and often protracted period of their lives in one interview, so it is perhaps inevitable that some details were omitted or compressed. There was also a variety in interviews, with some parents choosing to be interviewed alone, and others in couples. This inevitably means that some interviews are co-produced while others are not. There was little conflict in evidence during interviews although there were inevitable differences of opinion and recall.

CONCLUSION

As babies recover, the potential for parental expertise grows. Our study highlights the vital role of information, both about the condition and about the infant's progress. Health care staff could discuss or direct parents to information sources, seek to empower parents through involving them with their infant's care where possible, and link them with other parents.

A Contributorship statement The study was conceived by MK. All interviews were collected by LH. Analysis was undertaken by LH and LL, with input from MK and AM. LH

For peer review only

wrote the article, with input from the other authors. We thank the members of our parent advisory (PPI) group for their contributions throughout the study.

B Competing Interests Authors declare no competing interests

C Funding: This study was funded through a National Institute for Health Research (NIHR) Professorship award to Marian Knight (NIHR-RP-011-032). The views expressed are those of the author(s) and not necessarily those of the NHS, the NIHR or the Department of Health and by the National Institute for Health Research Biomedical Research Centre, Oxford, grant BRC-1215-20008 to the Oxford University Hospitals NHS Foundation Trust and the University of Oxford.

D Data Sharing statement Participants were invited to review their transcript and mark any sections that they did not want used before transferring copyright to the University of Oxford for use in research, teaching, publications and broadcasting. These carefully anonymised transcripts form part of a University of Oxford archive which is available to other bona fide research teams for secondary analysis. All authors had access to the all of the data in the study and take responsibility for the integrity of the data and the accuracy of the data analysis.

For peer review only

REFERENCES

1. Allin BSR, Tse WHW, Marven S, et al. Challenges of Improving the Evidence Base in Smaller Surgical Specialties, as Highlighted by a Systematic Review of Gastroschisis Management. *Plos One* 2015;10(1) doi: ARTN e0116908
10.1371/journal.pone.0116908
2. Allin B, Ross A, Marven S, et al. Development of a core outcome set for use in determining the overall success of gastroschisis treatment. *Trials* 2016;17 doi: ARTN 360
10.1186/s13063-016-1453-7
3. Lam HS, Sit T, Chau CL, et al. Attitudes of Parents and Health Care Workers to Major Surgery for High-Risk Preterm Infants. *The Journal of Pediatrics* 2016;177(Supplement C):78-83.e3. doi: https://doi.org/10.1016/j.jpeds.2016.06.071
4. Tam PKH, Chung PHY, St Peter SD, et al. Advances in paediatric gastroenterology. *Lancet* 2017;390(10099):1072-82.
5. Aagaard H, Hall EOC. Mothers' Experiences of Having a Preterm Infant in the Neonatal Care Unit: A Meta-Synthesis. *Journal of Pediatric Nursing* 2008;23(3):e26-e36. doi: https://doi.org/10.1016/j.pedn.2007.02.003
6. Wei H, Roscigno CI, Hanson CC, et al. Families of children with congenital heart disease: A literature review. *Heart Lung* 2015;44(6):494-511. doi: 10.1016/j.hrtlng.2015.08.005
7. Wigert H, Dellenmark MB, Bry K. Strengths and weaknesses of parent-staff communication in the NICU: a survey assessment. *Bmc Pediatr* 2013;13 doi: Artn 71
10.1186/1471-2431-13-71
8. Jordan B, Franich-Ray C, Albert N, et al. Early mother-infant relationships after cardiac surgery in infancy. *Arch Dis Child* 2014;99(7):641-5. doi: 10.1136/archdischild-2012-303488
9. Woolf-King SE, Anger A, Arnold EA, et al. Mental Health Among Parents of Children With Critical Congenital Heart Defects: A Systematic Review. *J Am Heart Assoc* 2017;6(2) doi: ARTN e004862
10.1161/JAHA.116.004862
10. Aite L, Bevilacqua F, Zaccara A, et al. Seeing Their Children in Pain: Symptoms of Posttraumatic Stress Disorder in Mothers of Children with an Anomaly Requiring Surgery at Birth. *Am J Perinat* 2016;33(8):770-75. doi: 10.1055/s-0036-1572543
11. Le Gouez M, Alvarez L, Rousseau V, et al. Posttraumatic Stress Reactions in Parents of Children Esophageal Atresia. *Plos One* 2016;11(3) doi: ARTN e0150760
10.1371/journal.pone.0150760
12. Kaasen A, Helbig A, Malt UF, et al. Acute maternal social dysfunction, health perception and psychological distress after ultrasonographic detection of a fetal structural anomaly. *BJOG* 2010;117(9):1127-38. doi: 10.1111/j.1471-0528.2010.02622.x
13. Connor JA, Kline NE, Mott S, et al. The Meaning of Cost for Families of Children With Congenital Heart Disease. *J Pediatr Health Car* 2010;24(5):318-25. doi: 10.1016/j.pedhc.2009.09.002
14. Froh EB, Deatrick JA, Curley MAQ, et al. Making Meaning of Pumping for Mothers of Infants With Congenital Diaphragmatic Hernia. *Jognn-J Obst Gyn Neo* 2015;44(3):439-49. doi: 10.1111/1552-6909.12564
15. Ainsworth MDB, M; Waters, E. . Patterns of attachment: a psychological study of the strange situation. New Jersey: Erlbaum 1978.

For peer review only

16. Bowlby J. The nature of the child's tie to the mother. *International Journal of Psychoanalysis* 1958;38(9):350-72.
17. Tregay J, Brown KL, Crowe S, et al. Signs of deterioration in infants discharged home following congenital heart surgery in the first year of life: a qualitative study. *Arch Dis Child* 2016;101(10):902-8. doi: 10.1136/archdischild-2014-308092
18. Lupton D, Fenwick J. 'They've forgotten that I'm the mum': constructing and practising motherhood in special care nurseries. *Soc Sci Med* 2001;53(8):1011-21. doi: Doi 10.1016/S0277-9536(00)00396-8
19. Wilkinson D. Death or Disability? The 'Carmentis Machine' and decision-making for critically ill children. Oxford: Oxford University Press 2013.
20. Kubota A, Yamakawa S, Yamamoto E, et al. Major neonatal surgery: psychosocial consequence of the patient and mothers. *J Pediatr Surg* 2016;51(3):364-67. doi: 10.1016/j.jpedsurg.2015.09.017
21. Laing S, McMahon C, Ungerer J, et al. Mother–child interaction and child developmental capacities in toddlers with major birth defects requiring newborn surgery. *Early Human Development* 2010;86(12):793-800. doi: https://doi.org/10.1016/j.earlhumdev.2010.08.025
22. Kratochvil MS, Robertson CMT, Kyle JM. Parents' View of Parent-Child Relationship Eight Years After Neonatal Intensive Care. *Social Work in Health Care* 1992;16(1):95-118. doi: 10.1300/J010v16n01_06
23. Huppatz K. Pierre Bourdieu: Health Lifestyles, the Family and Social Class. In: Collyer F, ed. Palgrave Handbook of Social Theory in Health, Illness and Medicine. Basingstoke: Palgrave Macmillan 2015:370-88.
24. Webb J, Schirato, T, Danaher, G, . Understanding Bourdieu. London: Sage 2002.
25. Locock L, Boylan, A-M., Snow, R., Staniszweska, S. The power of symbolic capital in patient and public involvement in health research. *Health Expectations* 2016 doi: DOI: 10.1111/hex.12519
26. Bourdieu P. What makes a social class? On the theoretical and practical existence of groups. *Berkeley J Sociol* 1987;32:1-17.
27. Bourdieu P. Distinction: A Critique of the Judgement of Taste. London: Routledge 1984.
28. Green JT, N. Qualitative Methods in Health Research. London: Sage 2004.
29. Glaser BS, Strauss A. The Discovery of Grounded Theory. New York: Adine Publishing 1967.
30. Green JT, Thorogood N. Qualitative Methods for Health Research. London: Sage 2004.
31. Pope C, Ziebland S, Mays N. Qualitative research in health care: Analysing qualitative data. *BMJ: British Medical Journal* 2000;320(7227):114.
32. Wei H, Roscigno CI, Swanson KM, et al. Parents' experiences of having a child undergoing congenital heart surgery: An emotional rollercoaster from shocking to blessing. *Heart & Lung: The Journal of Acute and Critical Care* 2016;45(2):154-60. doi: https://doi.org/10.1016/j.hrtlng.2015.12.007
33. Kosta L, Harms L, Franich-Ray C, et al. Parental experiences of their infant's hospitalization for cardiac surgery. *Child Care Hlth Dev* 2015;41(6):1057-65. doi: 10.1111/cch.12230
34. Gaskin KL, Barron DJ, Daniels A. Parents' preparedness for their infants' discharge following first-stage cardiac surgery: development of a parental early warning tool. *Cardiol Young* 2016;26(7):1414-24. doi: 10.1017/S1047951116001062

For peer review only - http://bmjopen.bmj.com/site/about/guidelines.xhtml

Table of Participants

Table 1 Participants

**Condition**	**Identifier**	**Parent Mother/Father**	**Sex of child**	**Child's age at interview**	**Intensive or high dependency care admission**
Exomphalos*	01	M	m	18 months	Yes
	02	M	f	6 years	Yes
	03	M	f	8 years	Yes
	04/05	M&F	f	8 years	Yes
	12/13	M&F	m	25 years	No
	16/17	M&F	m	13 years	Yes
	19	M	f	16 months	Yes
	41/42	M&F	m	Died age 7 months	Yes
Necrotising Enterocolitis	08	M	m	9 months	Yes
	09	M	m	11 months	Yes
	23	M	m	6 months	Yes
Hernia	26/27	M&F	f	9 months	No
Congenital Diaphragmatic Hernia*	10/11	M&F	m	9 months	Yes
	15	M	m	5 years	Yes
	24	M	m	9 years	Yes
Hirschprung's Disease*	18	M	f	1 year	No
	20/21	M&F	m	4 months	Yes
	22	M	m	16 months	No
	28/29	M&F	m	19 months	No
	39/40	M&F	m	5 years old	No
	43/44	M&F	m	5 years old	No
Gastroschisis*	30	M	m	7 ½ months	Yes
	31	M	m	4 years	Yes
	32	M	f	3 years	Yes
	33/34	M&F	m	3 months	Yes
	36	M	f	19 months	Yes
	37	M	f	6 years	Yes
Short Bowel	35	M	f	7 years	Yes
Jejunal atresia*	25	M	f	4 months	Yes

Oesophageal Atresia with trachea-oesophageal fistula*	38		m	19 months	Yes
Undiagnosed	14		m	6 months	
*Congenital diagnosis					

For peer review only

COREQ (COnsolidated criteria for REporting Qualitative research) Checklist

A checklist of items that should be included in reports of qualitative research. You must report the page number in your manuscript where you consider each of the items listed in this checklist. If you have not included this information, either revise your manuscript accordingly before submitting or note N/A.

Topic	Item No.	Guide Questions/Description	Reported on Page No.
**Domain 1: Research team and reflexivity**			
Personal characteristics			
Interviewer/facilitator	1	Which author/s conducted the interview or focus group?	p.8
Credentials	2	What were the researcher's credentials? E.g. PhD, MD	p.1 & 8
Occupation	3	What was their occupation at the time of the study?	p.1
Gender	4	Was the researcher male or female?	p.1
Experience and training	5	What experience or training did the researcher have?	p.1
Relationship with participants			
Relationship established	6	Was a relationship established prior to study commencement?	NO
Participant knowledge of the interviewer	7	What did the participants know about the researcher? e.g. personal goals, reasons for doing the research	p.8
Interviewer characteristics	8	What characteristics were reported about the inter viewer/facilitator? e.g. Bias, assumptions, reasons and interests in the research topic	p.8
**Domain 2: Study design**			
Theoretical framework			
Methodological orientation and Theory	9	What methodological orientation was stated to underpin the study? e.g. grounded theory, discourse analysis, ethnography, phenomenology, content analysis	p.6 & 9
Participant selection			
Sampling	10	How were participants selected? e.g. purposive, convenience, consecutive, snowball	p.8
Method of approach	11	How were participants approached? e.g. face-to-face, telephone, mail, email	p.8
Sample size	12	How many participants were in the study?	p.7
Non-participation	13	How many people refused to participate or dropped out? Reasons?	p.7
Setting			
Setting of data collection	14	Where was the data collected? e.g. home, clinic, workplace	p.8
Presence of non-participants	15	Was anyone else present besides the participants and researchers?	No
Description of sample	16	What are the important characteristics of the sample? e.g. demographic data, date	Table 1
Data collection			
Interview guide	17	Were questions, prompts, guides provided by the authors? Was it pilot tested?	No, but can be
Repeat interviews	18	Were repeat inter views carried out? If yes, how many?	No
Audio/visual recording	19	Did the research use audio or visual recording to collect the data?	p.8
Field notes	20	Were field notes made during and/or after the interview or focus group?	Yes
Duration	21	What was the duration of the inter views or focus group?	p.9
Data saturation	22	Was data saturation discussed?	p.9
Transcripts returned	23	Were transcripts returned to participants for comment and/or	.p.8

For peer review only

Topic	Item No.	Guide Questions/Description	Reported on Page No.
		correction?	
**Domain 3: analysis and findings**			
Data analysis			
Number of data coders	24	How many data coders coded the data?	p.9
Description of the coding tree	25	Did authors provide a description of the coding tree?	No
Derivation of themes	26	Were themes identified in advance or derived from the data?	p.9
Software	27	What software, if applicable, was used to manage the data?	p.9
Participant checking	28	Did participants provide feedback on the findings?	N/A
Reporting			
Quotations presented	29	Were participant quotations presented to illustrate the themes/findings? Was each quotation identified? e.g. participant number	P.10-17
Data and findings consistent	30	Was there consistency between the data presented and the findings?	Yes
Clarity of major themes	31	Were major themes clearly presented in the findings?	Yes
Clarity of minor themes	32	Is there a description of diverse cases or discussion of minor themes?	Yes

Developed from: Tong A, Sainsbury P, Craig J. Consolidated criteria for reporting qualitative research (COREQ): a 32-item checklist for interviews and focus groups. *International Journal for Quality in Health Care*. 2007. Volume 19, Number 6: pp. 349 – 357

Once you have completed this checklist, please save a copy and upload it as part of your submission. DO NOT include this checklist as part of the main manuscript document. It must be uploaded as a separate file.

For peer review only
